# Step-by-step optimization of a heterologous pathway for de novo naringenin production in *Escherichia coli*

**DOI:** 10.1007/s00253-024-13271-7

**Published:** 2024-08-10

**Authors:** Daniela Gomes, Joana L. Rodrigues, Ligia R. Rodrigues

**Affiliations:** 1 CEB-Centre of Biological Engineering, Universidade Do Minho, Campus de Gualtar, 4710-057 Braga, Portugal; 2 LABBELS - Associate Laboratory, Braga, Guimarães Portugal

**Keywords:** Naringenin, *Escherichia coli*, De novo biosynthesis, Synthetic biology, Heterologous production

## Abstract

**Abstract:**

Naringenin is a plant polyphenol, widely explored due to its interesting biological activities, namely anticancer, antioxidant, and anti-inflammatory. Due to its potential applications and attempt to overcome the industrial demand, there has been an increased interest in its heterologous production. The microbial biosynthetic pathway to produce naringenin is composed of tyrosine ammonia-lyase (TAL), 4-coumarate-CoA ligase (4CL), chalcone synthase (CHS), and chalcone isomerase (CHI). Herein, we targeted the efficient de novo production of naringenin in *Escherichia coli* by performing a step-by-step validation and optimization of the pathway. For that purpose, we first started by expressing two TAL genes from different sources in three different *E. coli* strains. The highest *p*-coumaric acid production (2.54 g/L) was obtained in the tyrosine-overproducing M-PAR-121 strain carrying TAL from *Flavobacterium johnsoniae* (*FjT*AL). Afterwards, this platform strain was used to express different combinations of 4CL and CHS genes from different sources. The highest naringenin chalcone production (560.2 mg/L) was achieved by expressing *Fj*TAL combined with 4CL from *Arabidopsis thaliana* (*At*4CL) and CHS from *Cucurbita maxima* (*Cm*CHS). Finally, different CHIs were tested and validated, and 765.9 mg/L of naringenin was produced by expressing CHI from *Medicago sativa* (*Ms*CHI) combined with the other previously chosen genes. To our knowledge, this titer corresponds to the highest de novo production of naringenin reported so far in *E. coli*.

**Key points:**

*• Best enzyme and strain combination were selected for de novo naringenin production.*

*• After genetic and operational optimizations, 765.9 mg/L of naringenin was produced.*

*• This de novo production is the highest reported so far in E. coli.*

**Supplementary Information:**

The online version contains supplementary material available at 10.1007/s00253-024-13271-7.

## Introduction

**Fig. 1:**
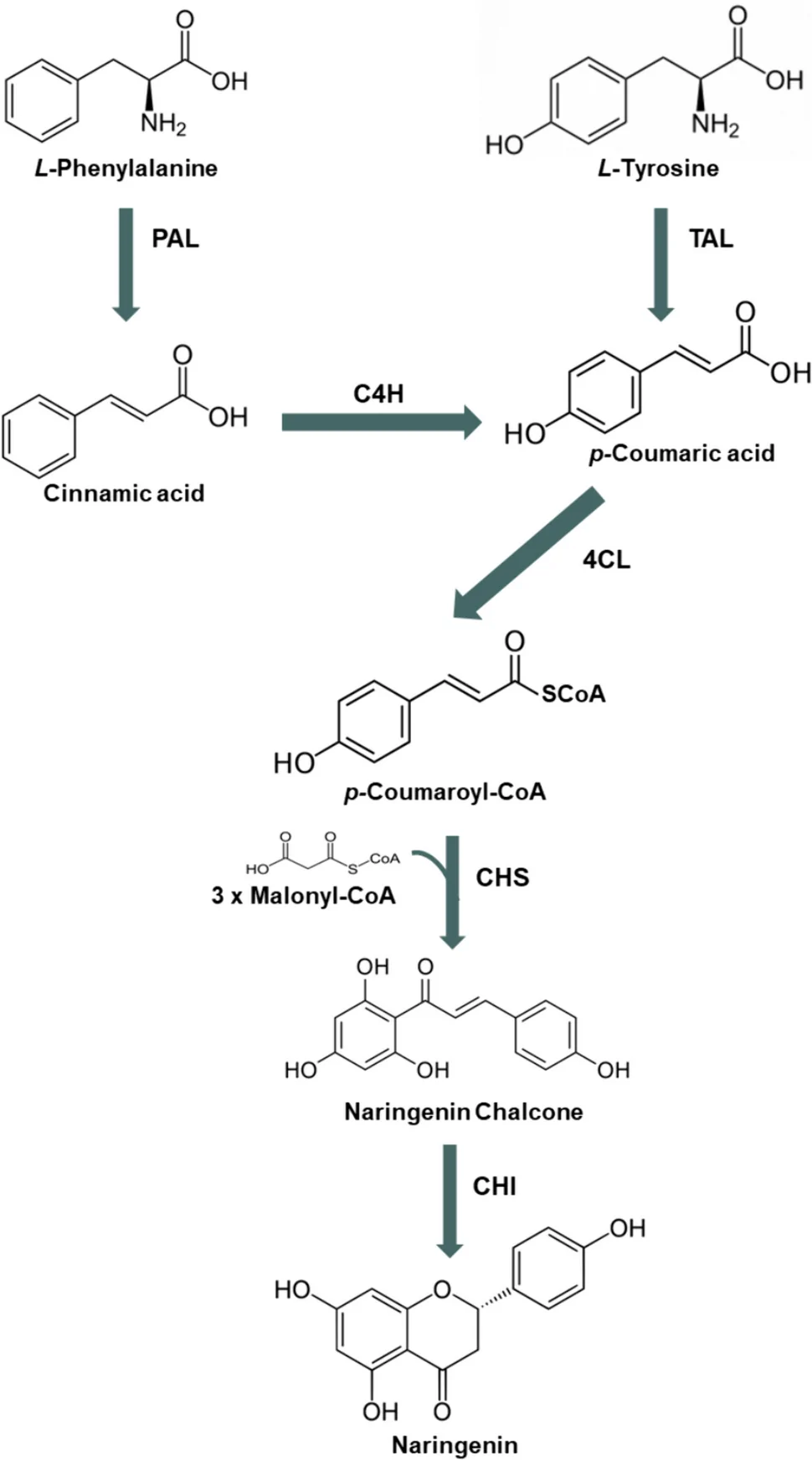
Naringenin biosynthetic pathway*.* Both *l*-phenylalanine and *l*-tyrosine can be used as starter substrates. The extender substrate of this pathway is malonyl-CoA. This pathway is composed of the following enzymes: 4-coumarate-CoA ligase (4CL), chalcone isomerase (CHI), chalcone synthase (CHS), cinnamate 4-hydroxylase (C4H), phenylalanine ammonia-lyase (PAL), and tyrosine ammonia-lyase (TAL)

Plants produce secondary metabolites, including polyphenols, as a defense mechanism to respond to biotic and abiotic stresses. Naringenin is a plant-derived polyphenolic compound that has several recognized biological activities, including antioxidant, anti-inflammatory, and anticancer (Ghofrani et al. 2015; Jin et al. 2017; Kataoka et al. 2020; Wadhwa et al. 2020; Shi et al. 2021). As other plant secondary metabolites, naringenin is produced and accumulated in very low amounts in plants, and its production is affected by climatic, seasonal, and geographical variations. Moreover, its extraction from native plants is considered unsustainable and difficult due to complex downstream processing that leads to low production yields. Additionally, the chemical synthesis is not considered an environmentally friendly and economic process to produce this compound as it uses toxic compounds and expensive substrates (Liu et al. 2017; Rainha et al. 2020; Gomes et al. 2022a, 2022b). Therefore, in the last years, the interest in the heterologous production of naringenin has increased to satisfy its industrial demand. The heterologous microbial production of naringenin requires the expression of several enzymes. Naringenin can be produced from the aromatic amino acids l-phenylalanine and l-tyrosine (Fig. 1) that are derived from the shikimate pathway. Through the l-phenylalanine route, the action of both phenylalanine ammonia-lyase (PAL) and cinnamic acid 4-hydroxylase (C4H) is essential to produce *p-*coumaric acid. Alternatively, when l-tyrosine is used as a substrate, only tyrosine ammonia-lyase (TAL) must be expressed to catalyze this conversion step. Then, *p-*coumaric acid is converted by 4-coumarate-CoA ligase (4CL) into *p-*coumaroyl-CoA being then converted into naringenin chalcone by chalcone synthase (CHS) using three molecules of malonyl-CoA as extender unit. Afterwards, chalcone isomerase (CHI) catalyzes the final conversion of naringenin chalcone into naringenin (Pandey et al. 2016; Yonekura-Sakakibara et al. 2019).

The biosynthetic pathway responsible for naringenin production has already been engineered in microbial chassis like *Escherichia coli*, *Yarrowia lipolytica*, *Saccharomyces cerevisiae*, and *Streptomyces* spp. (Koopman et al. 2012; Lv et al. 2019; Lyu et al. 2019; Dunstan et al. 2020; Palmer et al. 2020; Zhou et al. 2020b; Liu et al. 2021; Zhang et al. 2021; Ye et al. 2023)*.* In *E. coli*, the highest production of naringenin was reported by Zhou et al. (2020b) in a strain modified both to improve malonyl-CoA and l-tyrosine availability. In shake-flask and fed-batch reactor experiments, 485 mg/L and 585 mg/L of naringenin were produced, respectively. However, these production levels are not enough to reach an industrial production of naringenin using *E. coli* as a microbial host. Using *Y. lipolytica* as a host, Palmer et al. (2020) reported the production of naringenin in a malonyl-CoA-boosted strain. In test tube fermentations, 124.1 mg/L of naringenin was produced. This production was further improved to 357 mg/L and 898 mg/L in batch and fed-batch reactors, respectively (Palmer et al. 2020). As far as we know, the highest naringenin titer reported so far was achieved using a modified *S. cerevisiae* strain with improved malonyl-CoA availability (Zhang et al. 2021). In that study, 1129.44 mg/L of naringenin was obtained in a fed-batch bioreactor fermentation (Zhang et al. 2021). More recently, the heterologous production of naringenin in *Streptomyces albidoflavus* was reported. However, only 22.4 mg/L of naringenin was produced (Ye et al. 2023).

Despite the promising production levels achieved so far, several optimizations are still required to improve the production levels and achieve industrial scale. Beyond the titers, it is also important to have one process with high yields and productivity/rates. The optimization of these three metrics (titer, yield, and productivity/rates) is mandatory to achieve a scalable and cost-effective process at an industrial scale. The target values for these metrics vary depending on the specific compound produced (Wehrs et al. 2019; Olsson et al. 2022). In this study, we aimed to design, assemble, and validate an efficient pathway for de novo production of naringenin in *E. coli*. A step-by-step optimization was performed by testing different genes from different organisms and validated by performing production experiments and evaluating the production levels of the different intermediaries. The TAL step was also evaluated in three different *E. coli* strains to choose the best strain to reach higher production levels and to be further used for the validation of the next pathway steps. After choosing the best combination of the pathway enzymes and further performing optimizations in the production experiment parameters (i.e., time and carbon source concentration), 765.9 mg/L of naringenin was achieved corresponding to the highest de novo production level reported so far in *E. coli.*

## Materials and methods

### Strains, plasmids, and chemicals

*E. coli* NZY5α (NZYTech—MB00401) was used as a cloning and plasmid propagation strain. The expression and validation of the *p-*coumaric acid production pathway were performed in *E. coli* BL21 (DE3) (NZYTech—MB006), *E. coli* K-12 MG1655 (DE3) (Nielsen et al. 2010), and *E. coli* M-PAR-121 (Koma et al. 2020). The heterologous biosynthetic pathways to produce naringenin chalcone and naringenin were expressed in *E. coli* M-PAR-121. Table 1 shows the features of all the strains and plasmids that were used in this study. The plasmids pCBJ280, pBADMod1-CHS, pYS454, and pBbE2c-Gm4CL1 were kindly provided by Dr. Christian Bille Jendresen (Jendresen et al. 2015), Dr. Claudia Schmidt-Dannert (Watts et al. 2004), Dr. Toru Nakayama (Waki et al. 2020), and Dr. Mark Dunstan (Dunstan et al. 2020), respectively.

**Table 1:** Strains and plasmids used in this study

**Strains**	**Relevant genotype**	**Source**
* E. coli* NZY5	*fhuA2Δ(argF*^*−*^*lacZ)*U169 *phoA glnV44* Φ80 *Δ(lacZ)*M15 *gyrA96 recA1 relA1 endA1 thi-1 hsdR17*	NZYTech (MB00401)
* E. coli* BL21 (DE3)	F¯ *omp*T *gal dcm lon hsd*SB(rB- mB-) λ(DE3 *lacI lacUV5-T7 gene 1 ind1 sam7 nin5])	NZYTech (MB006)
* E. coli* K-12 MG1655 (DE3)	F¯ λ¯ *ilvG*¯ *rfb¯*50 *rph*¯1 λ(DE3)	Nielsen et al. (2010)
* E. coli* M-PAR-121	l-Tyrosine-overproducing strain derived from MG1655 (DE3); *tyrR*::P_T7lac_-*aroG*^*fbr*^* ldhA*::P_T7lac_-*tyrA*^*fbr*^* adhE*::P_T7lac_-*ppsA pflDC*::P_T7lac(-8TC)_-*tktA pykF*::P_T7lac_-*aroALC ascF*::P_T7lac_-*aroEDB*	Koma et al. (2020)
**Plasmids**	**Construct**	**Source**
pRSFDuet-1	RSF1030 ori, lacI, double PT7lac, Kan^R^	Novagen
pCDFDuet-1	CloDF13 ori, lacI, double PT7lac, Spec^R^	Novagen
pACYCDuet-1	P15A ori, lacI, double PT7lac, Cm^R^	Novagen
pCBJ280	F1 ori, AmpR; URA; pESC-URA-derived vector carrying codon-optimized tyrosine ammonia-lyase (TAL) from *Flavobacterium johnsoniae*	Jendresen et al. (2015)
pRSFDuet_*Rg*TAL	pRSFDuet-1 carrying codon-optimized TAL from *Rhodotorula glutinis*	Rodrigues et al. (2015b)
pACYCDuet_*At*4CL	pACYCDuet-1 carrying 4-coumarate-CoA ligase (4CL)-1 from *Arabidopsis thaliana*	Rodrigues et al. (2020)
pBbE2c-*Gm*4CL1	ColE1 ori, Cm^R^; pBbE2c vector carrying codon-optimized 4CL-1 from *Glycine max*	Dunstan et al. (2020)
pETM6-*Vv*4CL-*Cm*CHS-*Ms*CHI	ePathBrick expression vector, ColE1 ori, Amp^R^; pETM6 carrying codon-optimized 4CL from *Vitis vinifera*, codon-optimized chalcone synthase (CHS) from *Cucurbita maxima*, and chalcone isomerase (CHI) from *Medicago sativa*	Addgene #73,402 (Jones et al. 2016)
pETM6-*Pc*4CL-*Cm*CHS-*Ms*CHI	ePathBrick expression vector, ColE1 ori, Amp^R^; pETM6 carrying 4CL-2 from *Petroselinum crispum*, codon-optimized CHS from *C. maxima*, and CHI from *M. sativa*	Addgene #73,398 (Jones et al. 2016)
pETM6-*At*4CL-*Ph*CHS-*Cm*CHI	ePathBrick expression vector, ColE1 ori, Amp^R^; pETM6 carrying 4CL-1 from *A. thaliana*, codon-optimized CHS from *Petunia hybrida*, and CHI from *C. maxima*	Addgene #73,404 (Jones et al. 2016)
pBADMod1-CHS	pBAD/Thio-TOPO (Invitrogen)-derived vector, pUC ori, *araC*, Amp^R^; pBADMod1 holding CHS from *A. thaliana*	Watts et al. (2004)
pKYS454	pUC ori, Kan^R^, pENTR/D-TOPO-derived vector holding CHI from *A. thaliana*	Waki et al. (2020)
pRSFDuet_*Fj*TAL	pRSFDuet-1 carrying codon-optimized TAL from *F. johnsoniae*	This study
pACYCDuet_*Gm*4CL	pACYCDuet-1 carrying codon-optimized 4CL-1 from *G. max*	This study
pACYCDuet_*Vv*4CL	pACYCDuet-1 carrying codon-optimized 4CL from *V. vinifera*	This study
pACYCDuet_*Pc*4CL	pACYCDuet-1 carrying 4CL-2 from *P. crispum*	This study
pCDFDuet_*Cm*CHS	pCDFDuet-1 carrying codon-optimized CHS from *C. maxima*	This study
pRSFDuet_*Cm*CHS	pRSFDuet-1 carrying codon-optimized CHS from *C. maxima*	This study
pCDFDuet_*At*CHS	pCDFDuet-1 carrying CHS from *A. thaliana*	This study
pCDFDuet_*Ph*CHS	pCDFDuet-1 carrying codon-optimized CHS from *P. hybrida*	This study
pRSFDuet_*At*CHI	pRSFDuet-1 carrying CHI from *A. thaliana*	This study
pRSFDuet_*Ms*CHI	pRSFDuet-1 carrying CHI from *M. sativa*	This study
pRSFDuet_*Cm*CHI	pRSFDuet-1 carrying CHI from *C. maxima*	This study
pACYCDuet_*At*4CL (MCS2)	pACYCDuet-1 carrying 4CL-1 from *A. thaliana* (MCS2)	This study
pRSFDuet_*Fj*TAL_*Cm*CHS	pRSFDuet-1 carrying codon-optimized TAL from *F. johnsoniae* and CHS from *C. maxima*	This study
pRSFDuet_*At*4CL_*Cm*CHS	pRSFDuet-1 carrying 4CL-1 from *A. thaliana* and codon-optimized CHS from *A. thaliana*	This study
pACYCDuet_*Fj*TAL_*At*4CL	pACYCDuet-1 carrying codon-optimized TAL from *F. johnsoniae* and 4CL-1 from *A. thaliana*	This study
pACYCDuet_*At*4CL_*At*CHI	pACYCDuet-1 carrying 4CL-1 from *A. thaliana* and CHI from *A. thaliana*	This study
pACYCDuet_*At*4CL_*Ms*CHI	pACYCDuet-1 carrying 4CL-1 from *A. thaliana* and CHI from *M. sativa*	This study
pACYCDuet_*At*4CL_*Cm*CHI	pACYCDuet-1 carrying 4CL-1 from *A. thaliana* and CHI from *C. maxima*	This study

Lysogeny broth (LB) Miller medium and super optimal broth with catabolite repression (SOC) medium were purchased from NZYTech. Isopropyl β-d-1-thiogalactopyranoside (IPTG) used to induce protein expression was also purchased from NZYTech. *p-*Coumaric acid and l-tyrosine were obtained from Sigma-Aldrich, naringenin chalcone from Chengdu Biopurify Phytochemicals, and naringenin from Alfa Aesar. Glucose (Acros), NH_4_Cl (Panreac), NaCl (NZYTech), Na_2_HPO_4_ (Chem-Lab), KH_2_PO_4_ (Riel-deHaën), CaCO_3_ (Panreac), MgSO_4_ (Labkem), CaCl_2_ (Panreac), thiamine (Thermo Fisher Scientific), methionine (Panreac), nicotinic acid (Acros Organics), pyridoxine (Fisher BioReagents), biotin (Merck), folic acid (Panreac), riboflavin (Panreac), and pantothenic acid (Sigma-Aldrich) were used in the preparation of M9 minimal medium. For strain selection, the antibiotics kanamycin (NZYTech), ampicillin (VWR), chloramphenicol (NZYTech), and spectinomycin (Alfa Aesar) were used. For protein analysis, a sample buffer was prepared using Tris–HCl buffer pH 6.8 (Tris-base (Fisher Scientific) and HCl (VWR)), glycerol (Fisher Scientific), sodium dodecyl sulfate (SDS) (Fisher Scientific), bromophenol blue (Sigma-Aldrich), and β-mercaptoethanol (AppliChem). Coomassie Blue R-250 (Fisher Scientific) was used for gel staining. Ethyl acetate used for compound extraction from cell broth was purchased from Fisher Scientific. Acetonitrile, trifluoroacetic acid, and sulfuric acid, used in high-performance liquid chromatography (HPLC), were purchased from Fisher Scientific.

### Construction of plasmids

TAL from *Flavobacterium johnsoniae* (*Fj*TAL) was amplified by polymerase chain reaction (PCR) from pCBJ280 and cloned into pRSFDuet-1. 4CL-1 from *Glycine max* (*Gm*4CL) was amplified from pBbE2c-*Gm*4CL1. 4CL-2 from *Petroselinum crispum* (*Pc*4CL) was amplified from pETM6-*Pc*4CL-*Cm*CHS-*Ms*CHI. 4CL from *Vitis vinifera* (*Vv*4CL) was removed from pETM6-*Vv*4CL-*Cm*CHS-*Ms*CHI by restriction digestion with *Nde*I and *Xho*I enzymes. All the 4CL genes were subsequently cloned into the pACYCDuet-1 vector. Regarding the CHS genes, CHS from *Arabidopsis thaliana* (*At*CHS) was amplified by PCR from pBADMod1-CHS. CHS from *Petunia hybrida* (*Ph*CHS) was removed from the plasmid pETM6-*At*4CL-*Ph*CHS-*Cm*CHI by restriction digestion with *Nde*I and *Kpn*I enzymes. CHS from *Cucurbita maxima* (*Cm*CHS) was also removed from pETM6-*Vv*4CL-*Cm*CHS-*Ms*CHI by restriction digestion with the enzymes *Nde*I and *Xho*I. Both *Ph*CHS and *Cm*CHS were cloned into pCDFDuet-1. *Cm*CHS was also cloned into the pRSFDuet-1 vector. CHI from *A. thaliana* (*At*CHI), CHI from *Medicago sativa* (*Ms*CHI), and CHI from *C. maxima* (*Cm*CHI) were amplified from pKYS454 and pETM6-*Pc*4CL-*Cm*CHS-*Ms*CHI and pETM6-*At*4CL-*Ph*CHS-*Cm*CHI, respectively, and cloned into pRSFDuet-1. To construct pRSFDuet_*Fj*TAL_*Cm*CHS, *Cm*CHS was firstly cloned into the multiple cloning site (MCS) 2 of pRSFDuet-1, and *Fj*TAL was further cloned into the MCS1 of the constructed plasmid (pRSFDuet_*Cm*CHS). To construct pRSFDuet_*At*4CL_*Cm*CHS, 4CL from *A. thaliana* (*At*4CL) was amplified by PCR from pACYCDuet_*At*4CL and cloned into pRSFDuet_*Cm*CHS. To construct pACYCDuet_*Fj*TAL_*At*4CL, *At*4CL was cloned into the MCS2 of pACYCDuet-1. *Fj*TAL was further cloned into the MCS1 of the constructed plasmid. *At*CHI, *Ms*CHI, and *Cm*CHI were cloned into pACYCDuet_*At*4CL to construct pACYCDuet_*At*4CL_*At*CHI, pACYCDuet_*At*4CL_*Ms*CHI, and pACYCDuet_*At*4CL_*Cm*CHI, respectively. Tables S1 and S2 hold the sequences of all the genes and primers (Metabion/Eurofins) used, respectively.

NucleoSpin® Plasmid Miniprep Kit (Macherey–Nagel) was used for the isolation of plasmid DNA. All the genes were amplified by PCR using Phusion High-Fidelity DNA Polymerase (Thermo Scientific). The DNA fragments obtained were purified using the NucleoSpin® Gel and PCR Clean-up Kit from Macherey–Nagel. A NanoDrop One instrument (Thermo Scientific) was used to quantify the gene fragments and plasmid DNA. Afterwards, the fragments were digested using the appropriate restriction enzymes (Table S1) (Thermo Scientific) for 3 h at 37 °C. After digestion, NucleoSpin® Gel and PCR Clean-up Kit was used for their purification. Ligation reactions were performed at 22 °C for 1 h 30 min using T4 DNA ligase (Thermo Scientific). Ligation products were further transformed into *E. coli* NZY5α competent cells (NZYTech) by heat shock method. After transformation, the colonies were tested by colony PCR. Then, the plasmid was extracted and digested as a second confirmation step. Then, all the constructs were verified by DNA sequencing (Eurofins). After confirmation, the plasmids were transformed into chemically competent cells from the specific *E. coli* expression strain.

### Protein analysis

To confirm the expression of *Fj*TAL and all the 4CL, CHS, and CHI genes, *E. coli* M-PAR-121 carrying the desired plasmids were grown in LB Miller media at 37 °C. When an optical density at 600 nm (OD_600nm_) of 0.6 was reached (set as time 0 h), 10 mL of the sample was taken and centrifuged. Then, IPTG (final concentration of 0.1 mM) was added to the culture to induce the protein expression. The remaining culture was incubated for 6 h at 26 °C. At this time point (time 6 h), 10 mL of sample was also taken and centrifuged. The pellets were resuspended in 1 mL of Tris–HCl buffer (10 mM, pH 7.8). The cells were disrupted using a microtip probe linked to a Vibra-Cell processor (Sonics) by sonication on ice using the following conditions: 35% amplitude and 3 s ON plus 5 s OFF for a total of 7 min ON. Then, samples were centrifuged. The supernatant was recovered corresponding to the soluble phase. The pellet was resuspended in 1 mL of Tris–HCl buffer (10 mM, pH 7.8) corresponding to the insoluble phase. To quantify the amount of protein of both fractions, the Pierce Coomassie (Bradford) Protein Assay Kit was used following the manufacturer’s instructions. Protein fractions were mixed with 2 × sample buffer (65.8 mM Tris–HCl buffer pH 6.8, 26.3% glycerol, 2.1% SDS, 5% β-mercaptoethanol, and 0.01% bromophenol blue) and then denaturated at 100 °C for 5 min. Then, these fractions were subjected to SDS polyacrylamide gel electrophoresis (SDS-PAGE) to evaluate the expression levels. The SDS-PAGE gel was composed of 10% running gel and 4% stacking gel. The gel staining was performed using 0.2% (w/v) Coomassie Blue R-250 (Fisher Scientific) for 15 min. The gel was further destained with distilled water overnight. The protein ladders used were Color Prestained Protein Standard, Broad Range (10–250 kDa) (NEB) and NZYColour Protein Marker II (NZYTech).

### Metabolite production experiments

*E. coli* expression strain carrying different plasmids was cultivated overnight in LB Miller medium supplemented with the respective antibiotics, at 37 °C and 200 rpm. Then, 250-mL flasks containing 50 mL of LB Miller were inoculated using this preculture to a normalized initial OD_600nm_ of 0.1. The culture was incubated at 37 °C and 200 rpm until reaching an OD_600nm_ of 0.9. When this OD was achieved, the expression of the heterologous protein was induced by adding IPTG (final concentration of 0.1 mM). The culture was incubated at 26 °C during 5 h. Then, the cells were centrifuged at 8000 rpm for 5 min, and the pellet was resuspended in the M9 minimal medium. M9 minimal medium is composed by 3 g/L KH_2_PO_4_, 6 g/L Na_2_HPO_4_, 0.5 g/L NaCl, 1 g/L NH_4_Cl, 110 mg/L MgSO_4_, 15 mg/L CaCl_2_, 340 mg/L thiamine, 5 g/L CaCO_3_, and vitamins (12.2 mg/L nicotinic acid, 10.8 mg/L pantothenic acid, 2.8 mg/L pyridoxine, 0.84 mg/L riboflavin, 0.12 mg/L biotin, and 0.084 mg/L folic acid). M9 media was also supplemented with 40 g/L glucose, unless otherwise specified. l-Tyrosine (3 mM) was supplemented to the media when required. The M9 media was also supplemented with the required antibiotics (kanamycin (50 µg/mL), spectinomycin (100 µg/mL), and/or chloramphenicol (25 µg/mL) depending on the specific experiment). Unless otherwise specified, the production experiments were conducted in triplicate for 63 h. Supernatant samples (1 mL) were collected during the experiment. These samples were filtered and used to analyze *p-*coumaric acid and glucose. Whole broth samples (culture broth with cells—1 mL) were also collected to be further extracted and perform naringenin chalcone and naringenin analysis.

### Metabolite extraction

Since naringenin chalcone and naringenin are not completely diffused to the production medium and are mostly present inside of the *E. coli* cells, it was necessary to extract them from the cells to enable the analysis of these compounds. Whole broth (1 mL) was mixed with ethyl acetate (1:1 ratio). To achieve an efficient extraction of those compounds from the cells, the samples were vortexed for 2 min. Subsequently, the samples were centrifuged at 10,000 rpm for 2 min to separate the phases, and the organic phase (upper phase) was then transferred to a new tube. These steps of extraction were repeated until the pellet lost the yellow color (Rodrigues et al. 2020). The solvent was then evaporated in the fume hood to concentrate the extracts. The extracts were then resuspended in acetonitrile. The amount of acetonitrile added to the extracts varied between different samples and time points, ranging from 300 µL to 1 mL. For samples with very high production levels, additional dilution with acetonitrile was performed to fit within the calibration curve. Afterwards, the samples were analyzed by ultra-HPLC (UHPLC) (Rodrigues et al. 2020).

### Analytical methods

For glucose quantification, samples were analyzed by HPLC using a JASCO system associated with an RI detector (RI-2031). The column used to analyze the samples was the Aminex HPX-87H from Bio-Rad that was kept at 60 °C. The mobile phase used was 5 mM H_2_SO_4_ with a flow rate of 0.5 mL/min. Glucose was detected at a retention time of 10.9 min.

For *p-*coumaric acid, naringenin chalcone, and naringenin analysis, UHPLC was used. The chromatographic system was composed of a Kinetex® 2.6 µm Polar C18 100 Å LC column (150 × 4.6 mm) (Phenomenex) and the Shimadzu Nexera X2 system (Shimadzu Corporation, Kyoto, Japan) (LC-30AD pump unit, CBM-20A system controller, DGU20A 5R degasser unit, SIL-30AC autosampler unit, SPD-M20A detector unit, CTO-20AC column oven). Two mobile phases were used, namely 0.1% (v/v) of trifluoroacetic acid in water (mobile phase A) and acetonitrile (mobile phase B). Using a constant follow rate of 1 mL/min, the following gradient was used: 10–40% mobile phase B for 11 min and 40–70% for 2 min, 70–10% for 8 min and 10% mobile phase B for an additional 4 min. *p-*Coumaric acid was quantified based on the peak areas at 310 nm, and the retention time of the compound was 7.1 min. Naringenin chalcone and naringenin were detected at 290 nm, and the retention times were 12.0 min and 12.6 min, respectively.

### Statistical analysis

The results are presented as the mean value of three independent experiments ± standard deviation. Data statistical analysis was performed using GraphPad Prism Software, Inc., version 8.0.1. Ordinary one-way ANOVA tests were used to evaluate the statistical significance when required. The differences were considered significant when the *p-*value was < 0.05.

## Results

### Design and validation of an optimized heterologous *E. coli* strain to produce *p*-coumaric acid

In the phenylpropanoid pathway, both l-phenylalanine and l-tyrosine routes can be used to produce *p-*coumaric acid. l-Phenylalanine route requires the action of PAL and C4H enzymes. C4H is a plant membrane-bound cytochrome P450-dependent hydroxylase. These enzymes are difficult to express in bacteria since this microorganism lacks an endomembrane system, thus resulting in protein instability and insolubility (Watts et al. 2004; Gomes et al. 2022b). For this reason, the l-tyrosine route was chosen in this work to produce *p-*coumaric acid. Moreover, this route only requires the action of one enzyme to catalyze this step which is advantageous to reduce a possible metabolic burden imposed to the cells.

**Fig. 2:**
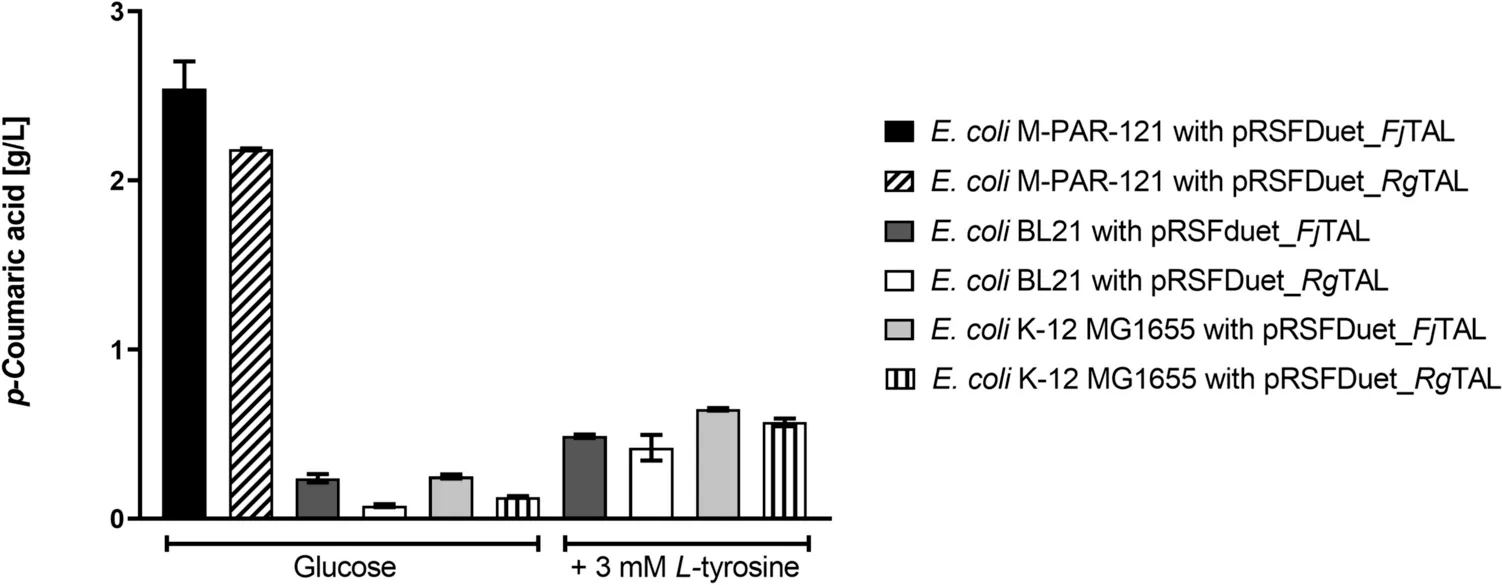
*p*-Coumaric acid production by *Escherichia coli* BL21 (DE3), K-12 MG1655 (DE3), and M-PAR-121 strains expressing tyrosine ammonia-lyase (TAL) gene from *Flavobacterium johnsoniae* (*Fj*TAL) or TAL gene from *Rhodotorula glutinis* *(*Rg*TAL).* The production experiments in *E. coli* M-PAR-121 were carried out using glucose as the sole substrate. The production experiments in *E. coli* BL21 (DE3) and K-12 MG1655 (DE3) were carried out in a first approach using glucose as the sole substrate and then with the supplementation of 3 mM *l*-tyrosine. Results correspond to the average of three independent experiments ± standard deviation

TAL from *Rhodotorula glutinis* (*Rg*TAL) and *Fj*TAL were selected to perform this first step of the biosynthetic pathway since they have been widely reported for the production of several polyphenolic compounds (Wu et al. 2014; Jendresen et al. 2015; Rodrigues et al. 2015a, 2020, 2022; Dunstan et al. 2020). Moreover, three different *E. coli* strains were tested to express both TAL genes: *E. coli* BL21 (DE3), *E. coli* K-12 MG1655 (DE3), and *E. coli* M-PAR-121 (Fig. 2). *E. coli* BL21 (DE3) and K-12 MG1655 (DE3) are common *E. coli* expression strains widely used in the biotechnology industry as platform strains to express the most diverse types of heterologous proteins (Castiñeiras et al. 2018). *E. coli* M-PAR-121 is a tyrosine-overproducing strain that has been derived from MG1655 (DE3) (Koma et al. 2020).

As presented in Fig. 2, the highest production levels of *p*-coumaric acid using glucose as substrate were obtained when the *E. coli* M-PAR-121 strain expressed either *Fj*TAL or *Rg*TAL. Moreover, as a proof of concept, a production experiment was carried out with supplementation of 3 mM of l-tyrosine to verify if the strains BL21 (DE3) and K-12 MG1655 (DE3) were able to achieve the same production levels obtained in M-PAR-121 without supplementation of l-tyrosine (Fig. 2). As can be observed in Fig. 2, the production of *p-*coumaric acid in both strains with supplementation of l-tyrosine did not reach the same production levels as in *E. coli* M-PAR-121 strain. However, higher production of *p*-coumaric acid was observed compared with the experiments performed with only glucose. Moreover, the production levels were slightly higher for the *E. coli* K-12 MG1655 (DE3) comparing with the ones achieved in *E. coli* BL21 (DE3). Regarding the expression of *Fj*TAL or *Rg*TAL, it was not possible to observe statistically significant differences in the production levels of *p-*coumaric acid in this production experiments with l-tyrosine supplementation.

The highest production of* p*-coumaric acid obtained in these experiments (2.54 g/L) was achieved for the *E. coli* M-PAR-121 strain expressing *Fj*TAL (Fig. 2). The expression of *Fj*TAL enzyme in *E. coli* M-PAR-121 strain was also validated through SDS-PAGE gel (Fig. S1). The production titer obtained in our study is equivalent to the one previously reported by Jones et al. (2017) (2.51 g/L). However, the productivity of our process was significantly higher compared with the one reported in their study (0.04 g/L/h vs 0.02 g/L/h). Considering these results, the M-PAR-121 strain expressing *Fj*TAL was chosen as the platform strain to construct and validate the following steps of the heterologous pathway (4CL, CHS, and CHI).

### Design and validation of an optimized heterologous *E. coli* strain to produce naringenin chalcone

**Fig. 3:**
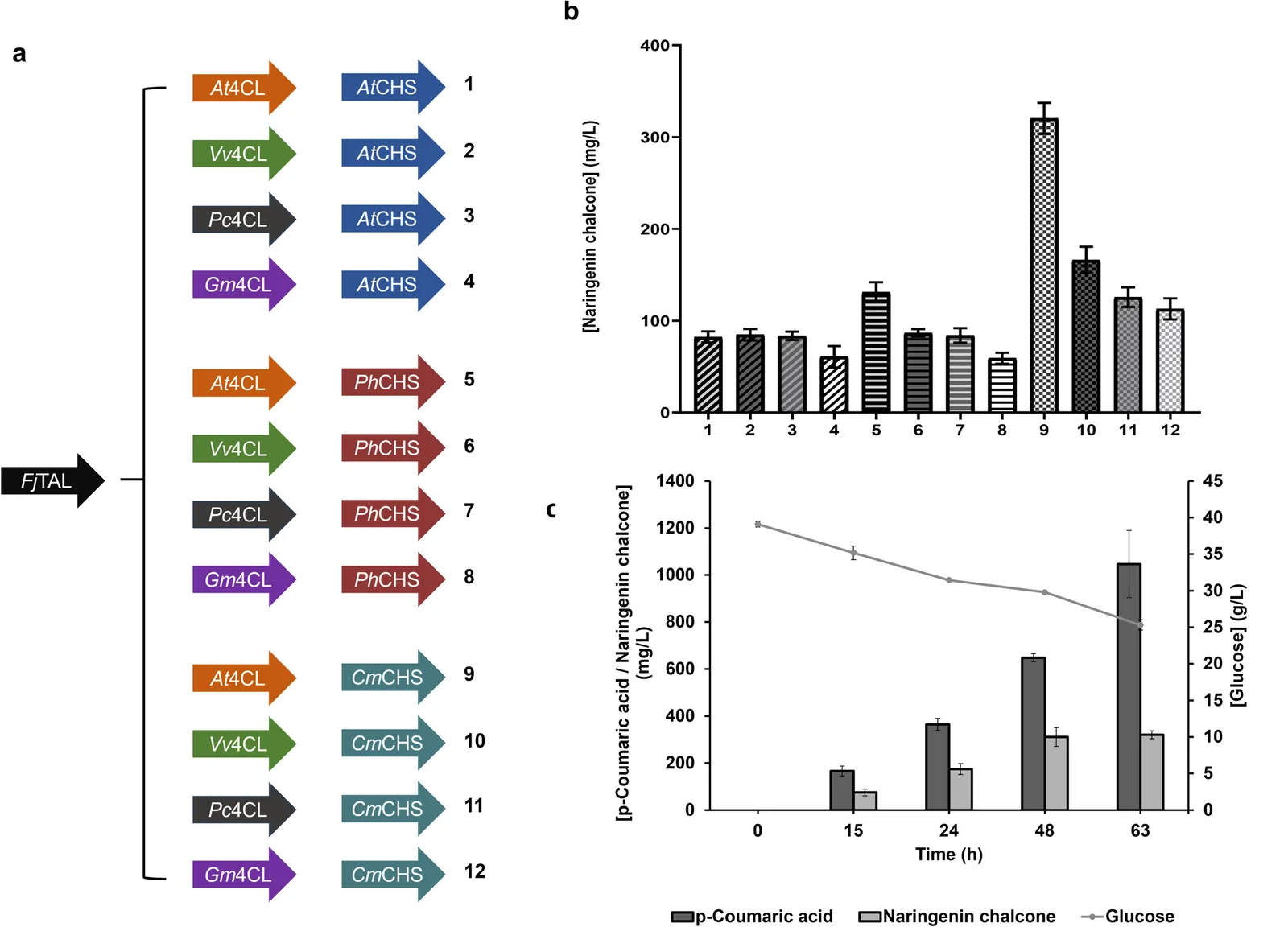
Naringenin chalcone production by *Escherichia coli* M-PAR-121 expressing 12 different biosynthetic pathways*.* **a** Schematic representation of the 12 different biosynthetic pathways*.* Tyrosine ammonia-lyase (TAL) gene from *Flavobacterium johnsoniae* (FjTAL) that was previously selected was expressed in the pRSFDuet-1 vector. The different 4-coumarate-CoA ligase (4CL) genes and the different chalcone synthase (CHS) genes were expressed in the pACYCDuet-1 vector and in the pCDFDuet-1 vector, respectively. **b** Naringenin chalcone production from 40 g/L of glucose by the 12 different constructed *E. coli *M-PAR-121 strains. **c** Profile of glucose consumption and *p*-coumaric acid and naringenin chalcone production for the *E. coli* M-PAR-121 strain expressing pRSFDuet_FjTAL, pACYCDuet_At4CL, and pCDFDuet_CmCHS (strain 9). The production experiments were maintained for 63 h. Results correspond to the average of three independent experiments ± standard deviation

After selecting the best *p*-coumaric acid producer, 4CL and CHS steps were constructed and validated to choose the best combination of enzymes to produce naringenin chalcone from glucose. *At*4CL, *Gm*4CL, *Vv*4CL, and *Pc*4CL were selected to test the conversion of *p-*coumaric acid into *p-*coumaroyl-CoA. *At*CHS, *Ph*CHS, and *Cm*CHS were selected to convert *p-*coumaroyl-CoA into naringenin chalcone. These genes were selected based on previous reports of heterologous production of naringenin and other polyphenols available in the literature (Xu et al. 2011; Wu et al. 2015; Jones et al. 2016; Dunstan et al. 2020; Rodrigues et al. 2020, 2022; Zhou et al. 2020b). The efficient individual expression of all these genes in *E. coli* M-PAR-121 was evaluated using SDS-PAGE (Figs. S2-S3). Then, considering the four different 4CL genes and the three different CHS genes, it was possible to construct 12 different pathway combinations (Fig. 3a).

These combinations were tested in the *E. coli* M-PAR-121 strain holding pRSFDuet_*Fj*TAL, and the production of naringenin chalcone using glucose as substrate was evaluated (Fig. 3b). After 63 h of fermentation, it was observed that the highest production of naringenin chalcone (320.4 mg/L) was obtained when *Fj*TAL, *At*4CL, and *Cm*CHS genes were expressed (strain 9 from Fig. 3a). Moreover, as can be observed in Fig. 3b, when the *Cm*CHS gene was expressed, a higher production of naringenin chalcone was obtained comparing directly with the other correspondent pathways (with the same 4CL gene and different CHS gene). The production profiles of *p-*coumaric acid and naringenin chalcone, as well as the profile of glucose consumption for strain 9, are presented in Fig. 3c. Regarding *p-*coumaric acid and naringenin chalcone production profiles, an increase in the titers of both products along the fermentation time was observed. Additionally, high amounts of *p-*coumaric acid were still accumulated at the end of fermentation (1046.9 mg/L). The same behavior was observed for the other strains (data not shown).

Regarding glucose, its concentration has decreased during the production experiment time, as expected. However, only 15 g/L of glucose was consumed, remaining almost 25 g/L of glucose at the end of the process.

**Fig. 4:**
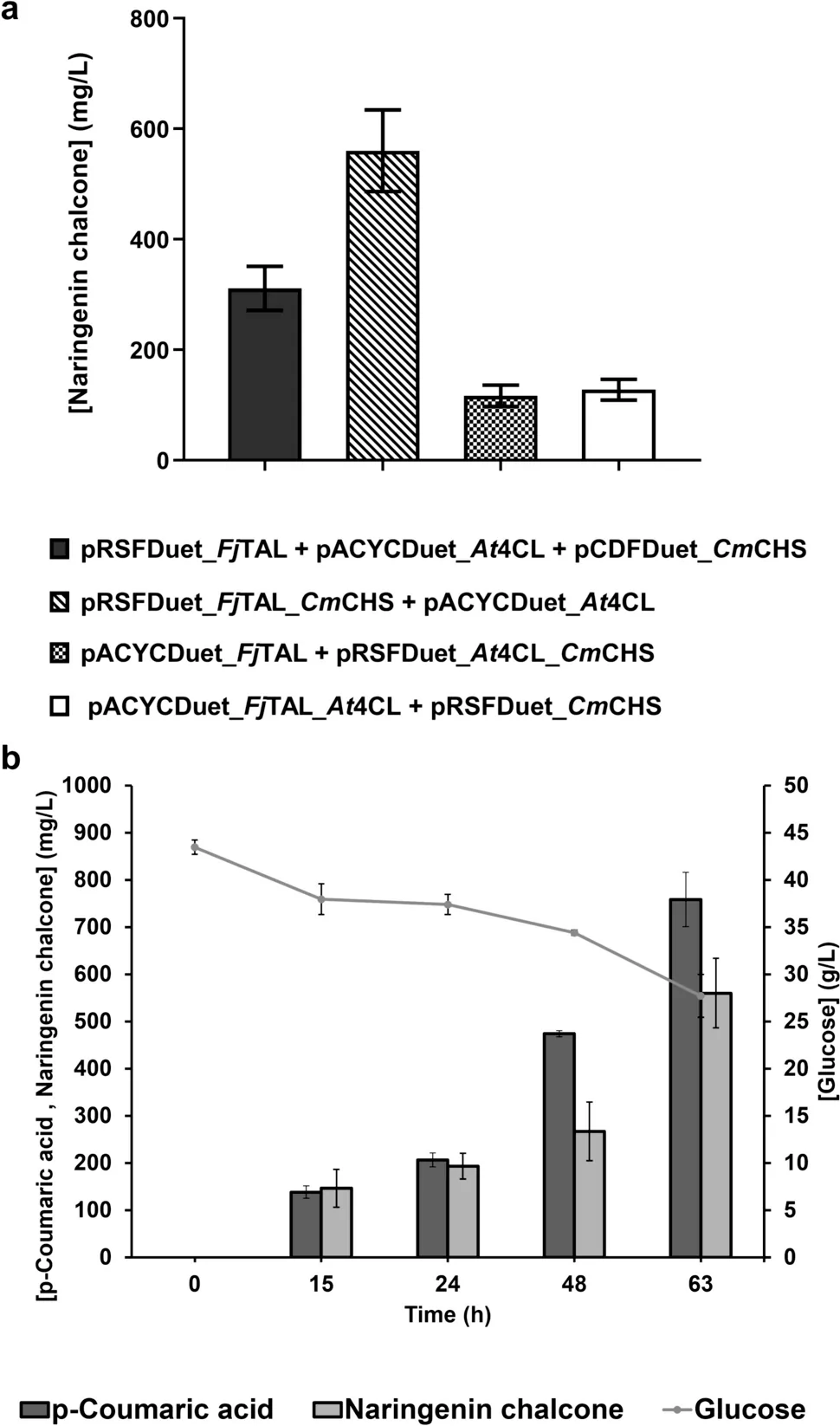
Evaluation of the effect on the production of naringenin chalcone of the reduction of cell’s metabolic burden. **a** Naringenin chalcone production from 40 g/L of glucose by the control strain (holding three plasmids) and by the three different *Escherichia coli* M-PAR-121 strains constructed to reduce the metabolic burden of the cells (holding two plasmids) after 63 h of fermentation. **b** Profile of naringenin chalcone and *p*-coumaric acid production, and glucose consumption for the *E. coli* M-PAR-121 carrying pRSFDuet_FjTAL_CmCHS and pACYCDuet_At4CL. Results correspond to the average of three independent experiments ± standard deviation

With the aim of improving the productivity of the constructed strain, a new strategy was designed to optimize the pathway balancing and to reduce the metabolic burden of the cells that can impair the production levels. This metabolic burden is imposed by the replication and maintenance of plasmids. The replication of plasmids and its maintenance implies the use of cell supplies, namely carbon building blocks and energy molecules (Silva et al. 2012; Rodrigues et al. 2020). Hence, the cell metabolic burden was reduced by cloning the three genes in only two plasmids instead of the three plasmids previously used. Considering that Duet plasmids (Novagen) have two multiple cloning sites containing two T7 promoters, two genes were cloned in one plasmid, and the other gene was cloned alone in another plasmid. The genes present in strain 9 were tested in different combinations in pRSFDuet-1 and pACYCDuet-1, and naringenin chalcone production was further evaluated (Fig. 4a).

Comparing with the control (strain expressing pRSFDuet_*Fj*TAL, pACYCDuet_*At*4CL, and pCDFDuet_*Cm*CHS), it is possible to conclude that only one strategy to reduce the metabolic burden of the cells (*E. coli* M-PAR-121 expressing pRSFDuet_*Fj*TAL_*Cm*CHS and pACYCDuet_*At*4CL) led to a significant improvement in the production of naringenin chalcone (≈1.7-fold improvement compared to the control). The other two strategies resulted in lower production levels of naringenin chalcone. Moreover, a lower accumulation of *p-*coumaric acid comparing to the control strain was observed during the production experiment (Fig. S4). The optimal constructed strain only consumed 16 g/L of glucose during the production experiment. Moreover, 560.2 mg/L of naringenin chalcone was produced. However, a significant amount of *p-*coumaric acid (758.8 mg/L) is still accumulated at the end of the experiment (Fig. 4b). The UHPLC chromatogram of this sample can be observed in Fig. S5.

### Design and validation of an optimized heterologous *E. coli* strain to produce naringenin

**Fig. 5:**
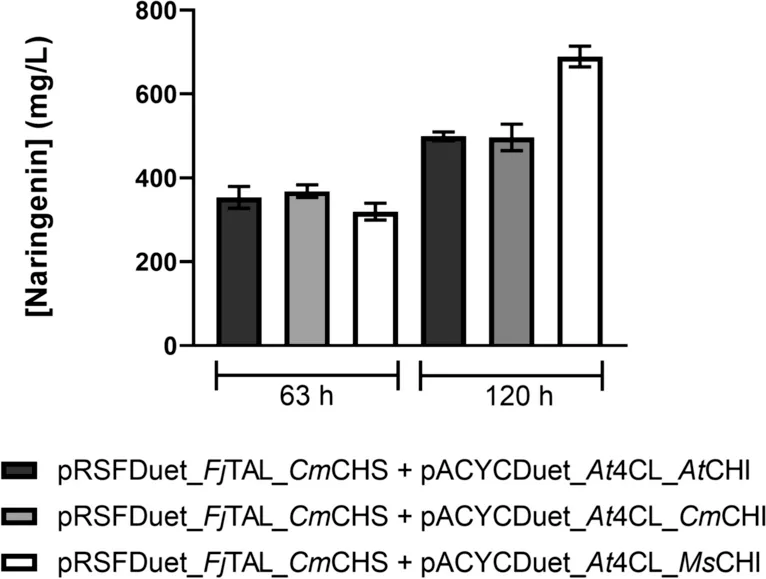
Naringenin production by *Escherichia coli* M-PAR-121 expressing three different biosynthetic pathways after 63 h and 120 h of fermentation*.* Results correspond to the average of three independent experiments ± standard deviation

CHI catalyzes the last step of the biosynthetic pathway responsible to produce naringenin. Several CHI genes derived from different species were tested in combination with *Fj*TAL, *At*4CL, and *Cm*CHS to optimize this step of the pathway and achieve the final production of naringenin from glucose. *At*CHI, *Ms*CHI, and *Cm*CHI were selected since they have been reported on the literature for the successful heterologous production of naringenin and also other flavonoids (Wu et al. 2015; Jones et al. 2016; Li et al. 2019; Wang et al. 2019; Dunstan et al. 2020; Zhou et al. 2020a). The expression of these genes in *E. coli* M-PAR-121 was individually tested and evaluated by SDS-PAGE gel (Fig. S6). Then, the CHI genes were cloned into the pACYCDuet_*At*4CL vector. The constructed vectors were expressed in the *E. coli* M-PAR-121 holding pRSFDuet_*Fj*TAL_*Cm*CHS. Production of naringenin from glucose was evaluated using the three constructed strains (Fig. 5).

At 63 h of fermentation, the three constructed strains produced 319.4 to 361.9 mg/L of naringenin, and no statistically significant differences between the tested pathways could be found. Furthermore, the naringenin production levels did not reach a plateau at 63 h, and high concentrations of *p-*coumaric acid were still available to be converted into the final product (Fig. S7). Considering that, the production experiment was prolonged until 120 h to also evaluate if there would be differences over time on the production levels for the different biosynthetic pathways tested (Fig. 5).

By expressing pRSFDuet_*Fj*TAL_*Cm*CHS and pACYCDuet_*At*4CL_*Ms*CHI plasmids, 689.5 mg/L of naringenin was produced at 120 h (Fig. 5). This production represents ≈2.2-fold production improvement comparing to the one obtained at 63 h, and a higher productivity was also obtained in this production experiment (5.8 mg/L/h vs 5.1 mg/L/h). The consumption of glucose and the profile of the production of *p-*coumaric acid and naringenin are presented in Fig. S8. Along the experiment, a high *p-*coumaric acid accumulation was observed similarly to what occurred on the other production experiments. Additionally, only 17 g/L of glucose was consumed during this production experiment by the *E. coli* M-PAR-121 strain expressing pRSFDuet_*Fj*TAL_*Cm*CHS and pACYCDuet_*At*4CL_*Ms*CHI.

**Fig. 6:**
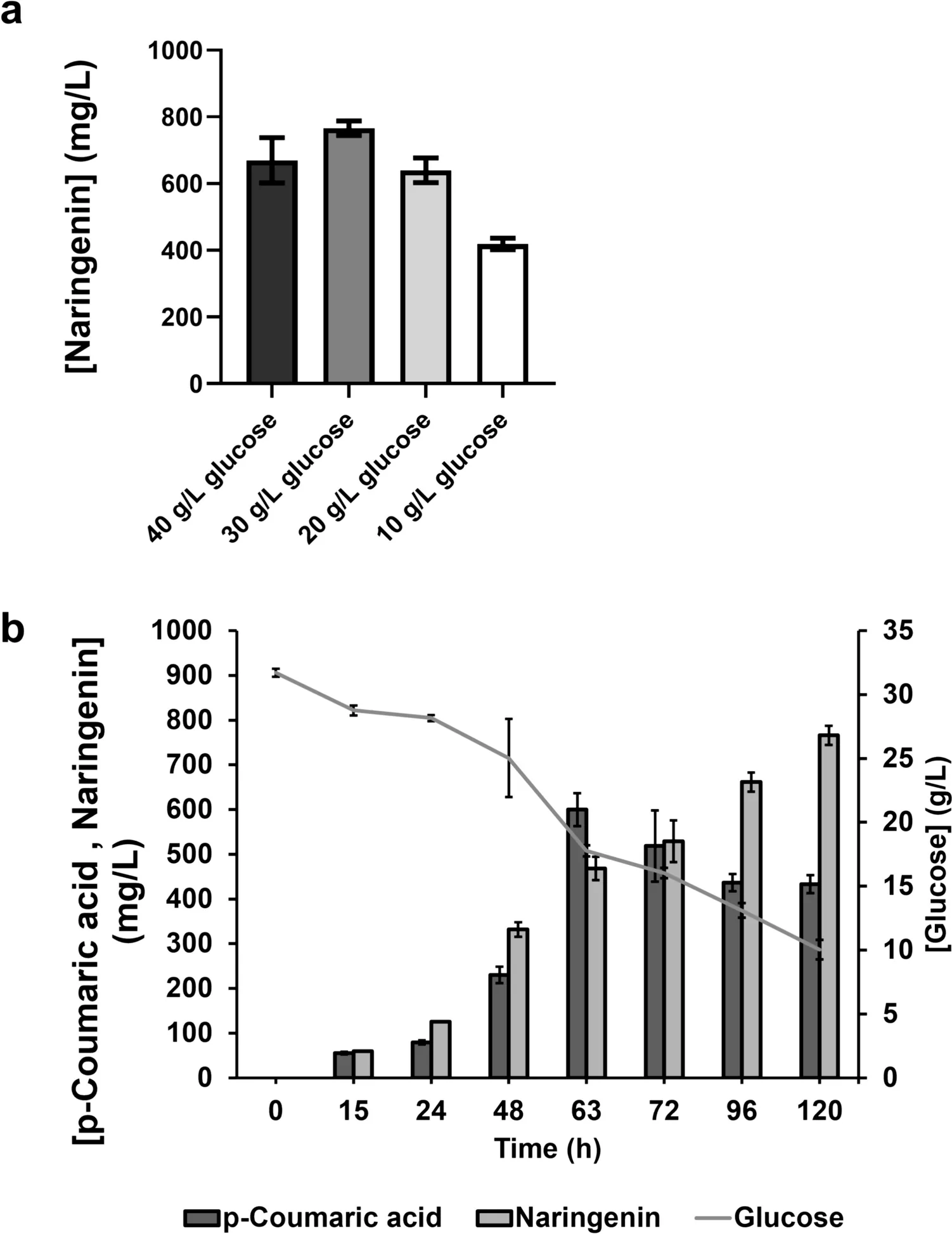
Evaluation of the effect of the carbon source on the levels of production of naringenin. **a** Naringenin production by *Escherichia coli* M-PAR-121 expressing the plasmids pRSFDuet_FjTAL_CmCHS and pACYCDuet_At4CL_MsCHI using different concentrations of glucose as carbon source (40 g/L, 30 g/L, 20 g/L, and 10 g/L). **b** Profile of naringenin and *p*-coumaric acid production and glucose consumption for the *E. coli* M-PAR-121 carrying pRSFDuet_FjTAL_CmCHS and pACYCDuet_At4CL_MsCHI when the production experiment was performed with 30 g/L of glucose. Results correspond to the average of three independent experiments ± standard deviation

After increasing the fermentation time, optimizations in the media components need to be performed to try to improve the production process. Since it was found that glucose accumulates at the end of all our production experiments, reducing glucose concentration was considered. Different concentrations of glucose were tested (40 g/L (control), 30 g/L, 20 g/L, and 10 g/L), and its effect on naringenin production was assessed (Fig. 6).

As shown in Fig. 6a, the highest production of naringenin was achieved when 30 g/L of glucose was used in the production experiment. Using this concentration, 765.9 mg/L of naringenin was produced, with a productivity of 6.4 mg/L/h and a yield of 0.03 g/g. This result represents a ≈1.1-fold improvement on the production levels compared to the control. The UHPLC chromatogram of this sample can be observed in Fig. S5. Beyond improving production levels, the use of a lower glucose concentration also has the advantage of reducing the production costs associated to the process. Additionally, it was possible to verify that significant *p-*coumaric acid amounts are still accumulated at the end of the production experiments (433.4 mg/L) and remain available to be subsequently converted into naringenin (Fig. 6b). Although the higher production titer and productivity were achieved using 30 g/L of glucose, higher production yields were reached using 20 g/L of glucose and 10 g/L of glucose as carbon source (0.03 g/g and 0.04 g/g, respectively). This naringenin production (765.9 mg/L) represents the highest de novo production of naringenin achieved so far in *E. coli*.

## Discussion

Naringenin is a plant polyphenolic compound with several known bioactivities and potential applications. Consequently, the industrial interest on this compound has been increasing, and the design of microbial cell factories able to produce this compound has been considered an alternative solution to reach its industrial production. By performing a step-by-step validation and optimization of the biosynthetic pathway, we have constructed an optimized *E. coli* strain able to produce *de novo* naringenin with the highest production levels reported so far in the literature using *E. coli* as a microbial chassis. This step-by-step optimization started by the validation of the first step of the biosynthetic pathway, catalyzed by TAL, to produce *p-*coumaric acid. Herein, two different TAL genes (*Rg*TAL or *Fj*TAL) were expressed in three different *E. coli* strains (Fig. 2). From the three tested strains, *E. coli* M-PAR-121 was the one that was able to produce the highest *p-*coumaric acid amounts. This strain was previously engineered to boost the l-tyrosine production pool. Therefore, it can produce high amounts of l-tyrosine from glucose being available to be converted into *p*-coumaric acid. This strain was constructed by Koma et al. (2020) by integrating two genes of the central metabolic pathway and eight genes of the shikimate pathway. Moreover, it is a plasmid-free strain which facilitates its use for the construction of metabolic pathways, and it was able to produce twofold l-tyrosine comparing with other previously constructed l-tyrosine plasmid-free overproducing strains (Koma et al. 2020). For all the strains, the highest production levels of *p*-coumaric acid were achieved when *Fj*TAL was expressed. This was expected since *Fj*TAL has a lower *K*_*m*_ than *Rg*TAL for l-tyrosine (6.7 µM vs 380 µM), as reported by Jendresen et al. (2015) and Zhou et al. (2016). *Fj*TAL has also a very low ability to accept l-phenylalanine comparing with its ability to accept l-tyrosine with a *K*_cat_/*K*_m_ ratio of 2400 (Jendresen et al. 2015). In contrast, *Rg*TAL has only *K*_cat_/*K*_m_ ratio of 10 (Zhou et al. 2016). These catalytic properties demonstrate that the *Fj*TAL enzyme is highly specific to accept tyrosine and has a high catalytic efficiency (Jendresen et al. 2015; Zhou et al. 2016; Haslinger and Prather 2020). Moreover, it was also recently shown by Virklund et al. (2023) that *Fj*TAL has a low affinity for *p-*coumaric acid being less prone to suffer product inhibition. To verify if *E. coli* K-12 MG1655 (DE3) and *E. coli* BL21 (DE3) can reach the same production levels than *E. coli* M-PAR-121, l-tyrosine was supplemented to the production media. Although the production of *p-*coumaric acid has increased comparing with the production levels achieved in the experiments performed with only glucose, the production on both strains did not reach the same production levels that were achieved by the *E. coli* M-PAR-121 without any supplementation of l-tyrosine (Fig. 2). This result reinforces that M-PAR-121 has a high pool of l-tyrosine being produced due to the modifications performed in its central metabolism that is consequently available for *p-*coumaric acid production. In fact, the *E. coli* M-PAR-121 strain was reported to be able to produce 2.2 g/L of tyrosine that corresponds to 12.1 mM (Koma et al. 2020). Considering this endogenous production of tyrosine, the supplementation of 3 mM of l-tyrosine in the BL21 (DE3) and K-12 MG1655 (DE3) strains did not meet the levels of endogenous production of the M-PAR-121 strain. This discrepancy may explain why these strains could not reach the production levels achieved in the M-PAR-121 strain. This l-tyrosine concentration (3 mM) was chosen based on previous reports of heterologous production of hydroxycinnamic acids and other polyphenols in *E. coli* (Katsuyama et al. 2008; Rodrigues et al. 2015a, 2020; Haslinger and Prather 2020). The supplementation of 12 mM of l-tyrosine to match the amount produced by the M-PAR-121 strain would not be reasonable, as this substrate is expensive and would increase the costs associated with the production process. Nevertheless, since the main goal of our work was to achieve the final production of naringenin without supplementing expensive precursors, the *E. coli* M-PAR-121 strain expressing *Fj*TAL was selected as platform strain to test the next steps of the pathway. Moreover, comparing *Fj*TAL and *Rg*TAL efficiency in these production experiments with l-tyrosine supplementation, it was not possible to observe differences statistically significant in the production levels. This result demonstrates that both TAL enzymes have a good performance when l-tyrosine concentrations are not limiting, which has been previously reported (Haslinger & Prather (2020)).

After choosing this strain, 4CL and CHS steps were validated by testing genes from different organisms (Fig. 3). Regarding 4CL, our study showed higher production levels when *At*4CL was expressed. Similarly, Jones et al. (2016) reported higher productions of naringenin and eriodyctiol in all the pathways holding the *At*4CL gene independently of the CHS and CHI expressed. Moreover, regarding the 4CL genes from *A. thaliana* identified so far (Ehlting et al. 1999), the isoform used in our study (4CL1) holds a low *K*_m_ for *p*-coumaric acid, showing its specificity for this substrate. Notwithstanding, the 4CL step is always considered a critical step in the phenylpropanoid pathway. This enzyme catalyzes the conversion of *p-*coumaric acid into *p-*coumaroyl-CoA in two steps. The first step corresponds to the adenylate formation and the second one to the thioester formation. These two steps require the presence of adenosine triphosphate (ATP), coenzyme A (CoA), and Mg^2+^. Since these molecules are also used by key metabolic pathways, it is expected that they are less available to be used on the phenylpropanoid pathway, possibly being a limiting factor to achieve higher production levels (Ehlting et al. 2001; Lavhale et al. 2018). Moreover, 4CL enzymes are not completely efficient on the conversion of the hydroxycinnamic acid to its derived CoA ester (Ehlting et al. 1999, 2001; Lavhale et al. 2018). Considering that, improving their activity through the construction of mutant versions of the 4CL enzyme can be an interesting approach to be employed. For example, Xiong et al. (2017) have designed a random mutagenesis library of the *At*4CL1 and have found one mutant with a 1.7-fold higher catalytic efficiency for *p-*coumaric acid comparing to the wild-type enzyme, leading also to a significant improvement in the heterologous production of resveratrol and naringenin. This strategy can also be considered in the future to improve the production levels (Xiong et al. 2017). Regarding the CHS step, the highest productions were achieved when *Cm*CHS was expressed (Fig. 3b). This result demonstrates the higher efficiency of this gene to convert *p*-coumaroyl-CoA into naringenin chalcone. Moreover, it also suggests that *Cm*CHS is more efficient than the other tested CHS genes to compete with the primary metabolism pathways that use malonyl-CoA, namely the fatty acid biosynthesis pathway.

After the identification of the best pathway enzymes to produce naringenin chalcone, strategies to reduce the metabolic burden of the cells and to balance the pathway were tested. Only one of these strategies led to an increase in the production levels (Fig. 4). In this strategy (*E. coli* M-PAR-121 expressing pRSFDuet_*Fj*TAL_*Cm*CHS and pACYCduet_*At*4CL), the *Fj*TAL gene was maintained on a high-copy plasmid, resulting in higher *p-*coumaric acid production during the experiment. The *At*4CL gene remained in its original plasmid backbone (pACYCDuet-1). Additionally, the *Cm*CHS gene was transferred from a medium-copy plasmid (pCDFDuet-1) to a high-copy plasmid (pRSFDuet-1), likely enhancing the conversion of *p-*coumaroyl-CoA to naringenin chalcone. In the other two strategies, the *Fj*TAL gene was moved to a low-copy plasmid (pACYCDuet-1). The rationale behind these strategies was to reduce *p-*coumaric acid accumulation and observe its impact on the production of the final compound. As anticipated, there was a lower accumulation of *p-*coumaric acid during the experiment, which seems to have limited naringenin chalcone production. Moreover, in both strategies, the *At*4CL gene was moved to the pRSFDuet-1 vector (pRSFDuet_*At*4CL_*Cm*CHS) and to the MCS2 of the pACYCDuet-1 vector (pACYCDuet_*Fj*TAL_*At*4CL). Previous research performed by our group also found lower curcumin production when the *At*4CL gene was moved from the pACYCDuet-1 vector to the pRSFDuet-1 vector (Rodrigues et al. 2020). This finding supports the idea that maintaining *At*4CL in MCS1 of pACYCDuet-1 is critical for the efficient conversion of *p-*coumaric acid to *p-*coumaroyl-CoA (Rodrigues et al. 2020).

Naringenin chalcone has been previously demonstrated to spontaneously cyclize to form naringenin (Mol et al. 1985). However, it was found that this self-cyclization mostly occurs under basic conditions, with a higher cyclization rate at a pH of 7.5. Moreover, self-cyclization is dramatically reduced at pH values ≤6.5, with only a 10% rate of self-cyclization observed at these pH values (Mol et al. 1985). In our experiment, the initial pH of the M9 media is 6.5, maintained constant throughout the production experiment due to the presence of CaCO_3_ in suspension, which is added for pH maintenance. CaCO_3_ is commonly used in acid-forming microbial processes to maintain the pH at around 6.5 (Salek et al. 2015; Ronoh et al. 2022). Therefore, significant cyclization is not expected to be observed in our experiment. This could be confirmed by UHPLC. The representative chromatogram of the naringenin chalcone production experiment (Fig. S5) shows a clear peak corresponding to naringenin chalcone at 12.0 min and only a very small peak at 12.6 min that corresponds to the naringenin retention time. Since the peak corresponding to naringenin in the samples is very small, the self-cyclization of naringenin chalcone into naringenin was considered negligible. This lack of significant cyclization demonstrates that the expression of CHI is essential for converting naringenin chalcone to naringenin and completing the pathway. Consequently, the CHI step was evaluated through the expression of three different CHI from different origins (Fig. 5). After increasing fermentation time, *Ms*CHI was found to be more efficient leading to a higher production of naringenin. Similarly, Leonard et al. (2007) reported higher amounts of naringenin, pinocembrin, and eriodyctiol by expressing *Ms*CHI instead of other CHI not compared here (e.g., CHI from *P. hybrida*) (Leonard et al. 2007). After testing different glucose concentrations, 765.9 mg/L of naringenin was produced (Fig. 6). During the naringenin production experiments, only the naringenin peak was detected by UHPLC demonstrating that *Ms*CHI was highly efficient to convert naringenin chalcone into naringenin (Fig. S5). The fact that only one peak was found in the samples of the naringenin production experiments supports the previous finding that CHI activity to catalyze the naringenin chalcone cyclization is 10^7^-fold more efficient than self-cyclization (Cheng et al. 2018).

Compared to previous reports in the literature, the naringenin production herein obtained (765.9 mg/L) is a great achievement in *E. coli* being the highest reported so far using this microorganism. The highest production previously reported was 485 mg/L in shake flask experiments and 585 mg/L in a fed-batch bioreactor using an *E. coli* strain modified both to improve tyrosine and malonyl-CoA flux (Zhou et al. 2020a). Comparing with the production achieved in *Y. lipolytica* (898 mg/L), the productivity of our production process was significantly higher (6.4 mg/L/h vs 3.0 mg/L/h) (Palmer et al. 2020). Beyond *Y. lipolytica*, the highest naringenin titer reported so far was achieved in *S. cerevisiae* strain (Zhang et al. 2021). Zhang et al. (2021) modified a previously constructed platform *S. cerevisiae* strain holding the naringenin pathway to improve its malonyl-CoA flux and have compared the production levels between the modified and non-modified strain. The non-modified strain was able to produce 703.5 mg/L of naringenin. In contrast, the modified strain was able to produce 1129.4 mg/L. This difference in the production levels between both strains suggests that improving malonyl-CoA levels is necessary to increase the final production of naringenin. However, these production levels were achieved at a fed-batch bioreactor scale being not possible to compare with the production levels obtained in our study. In our work, we have performed one extensive step-by-step optimization of the biosynthetic pathway. Several genes from different sources were tested to achieve the highest production levels in each step. We believe that this step-by-step optimization allowed us to find a combination of genes able to produce high amounts of naringenin. As far as we know, this is the first report of naringenin production using this specific combination of genes of the pathway (*Fj*TAL, *At*4CL, *Cm*CHS, and *Ms*CHI). Beyond the extensive exploitation of different genes for each step, we have also tested different *E. coli* strains. The production levels of one of the key intermediates of the pathway (*p-*coumaric acid) were significantly higher in the *E. coli* M-PAR-121. The utilization of this strain able to produce high levels of l-tyrosine can also be an advantage comparing with previous reports of heterologous production of naringenin in *E. coli*. We believe that the combination of the new pathway genes and the use of a highly efficient tyrosine-overproducing strain is the main reason for our high production levels. This highlights the importance of testing different chassis and pathway genes to find the optimal combination. Although we achieved these production levels without improving the malonyl-CoA availability, we believe that this factor can also be a rate-limiting step in our work hampering higher production levels of naringenin since three molecules of this compound are required in this pathway to perform the extension reaction (Fig. 1). Malonyl-CoA is naturally synthesized by *E. coli* and participates in key metabolic processes, namely in the production of phospholipids and fatty acids. Moreover, its synthesis is tightly regulated, and it is present at low levels inside of the *E. coli* cells, which limits its utilization on the biosynthetic pathways responsible for the production of heterologous compounds (Milke and Marienhagen 2020). Several different strategies have already been used to increase malonyl-CoA availability in engineered strains and successfully improve the production levels of naringenin (Xu et al. 2011; Wu et al. 2014, 2015; Zhou et al. 2020b, 2020a). In the future, it will be interesting to improve the malonyl-CoA synthesis by overexpressing key genes involved in its synthesis, for example, the malonate assimilation pathway genes *matB* and *matC* from *Rhizobium trifolii* and the native acetyl-CoA carboxylase to improve malonate and acetyl-CoA conversion into malonyl-CoA, respectively (Xu et al. 2011; Wu et al. 2014). Another interesting alternative that can be considered is the overexpression of the native pyruvate dehydrogenase and phosphoglycerate kinase genes to improve the synthesis of the acetyl-CoA intermediate (Xu et al. 2011; Wu et al. 2014). The deletion of genes of the tricarboxylic acid cycle and glycolysis should also be considered, namely the knockout of genes corresponding to fatty acids synthases (*fabB* and *fabF*), fumarase, succinyl-CoA synthetase, and acetaldehyde dehydrogenase (Wu et al. 2015). The combination of these overexpression and deletion strategies should be tested in the future in our strain to evaluate the production levels.

In conclusion, by performing a step-by-step optimization, *E. coli* M-PAR-121 expressing pRSFDuet_*Fj*TAL_*Cm*CHS and pACYCDuet_*At*4CL_*Ms*CHI was selected as the best producer of naringenin (769.5 mg/L–6.4 mg/L/h) (Fig. 6). To our knowledge, this is the first time that this optimized combination of genes was used to produce naringenin. Moreover, this naringenin production level corresponds to the highest production reported so far using *E. coli.* However, there is still a long way to go to achieve the production of this compound at an industrial scale, since a higher titer, yield, and productivity should be required to attain a cost-effective industrial production process. To optimize the metabolic flux of the pathway in the future, considerations should include improving the efficiency of the 4CL step and optimizing CoA and malonyl-CoA availability at the genetic level. Optimizations at the operational conditions level, namely the use of a single production media instead of using the combination of LB with M9, should also be considered to further implement a production process at a larger scale.

## Supplementary Information

Below is the link to the electronic supplementary material.

Supplementary file1 (PDF 1056 KB)

## Data Availability

The data supporting the findings of this study is available within the article and its supplementary material.
