# Supplementary material for: Step-by-step optimization of a heterologous pathway for de novo naringenin production in Escherichia coli
Source: Appl Microbiol Biotechnol. 2024 Aug 10;108(1):435. doi: 10.1007/s00253-024-13271-7 (PMC11316701; doi:10.1007/s00253-024-13271-7)
Supplement: Supplementary file 1 — Supplementary file1 (PDF 1056 KB) [file 253_2024_13271_MOESM1_ESM.pdf]

Applied Microbiology and Biotechnology

Supplementary material

Step-by-step optimization of a heterologous pathway for *de novo* naringenin production in *Escherichia coli*

Daniela Gomes,[1*] Joana L. Rodrigues,[1,2*] Ligia R. Rodrigues [1,2]

[1] CEB-Centre of Biological Engineering, Universidade do Minho, Campus de Gualtar, 4710-057, Braga, Portugal

[2] LABBELS- Associate Laboratory, Braga/Guimarães

* Corresponding author: joanarodrigues@deb.uminho.pt; Phone: +351 253 601 970

Table S1. Sequence of all the genes used in this study.

Gene	Sequence
***Rg*TAL**	ATGGCTCCGCGTCCGACCTCGCAATCCCAAGCTCGCACCTGCCCGACCACCCAAGTTACC<br>CAAGTTGACATCGTTGAAAAAATGCTGGCGGCGCCGACCGATTCGACGCTGGAACTGGAC<br>GGCTATAGCCTGAACCTGGGTGATGTGGTTTCTGCAGCACGTAAAGGTCGTCCGGTGCGT<br>GTTAAAGATTCAGACGAAATTCGCTCGAAAATCGATAAAAGCGTGGAATTTCTGCGTAGC<br>CAGCTGAGCATGTCTGTTTACGGCGTCACCACGGGTTTCGGCGGTTCAGCCGATACCCGC<br>ACGGAAGACGCCATTTCGCTGCAGAAAGCACTGCTGGAACATCAACTGTGCGGCGTGCTG<br>CCGAGCTCTTTTGATAGCTTCCGCCTGGGCCGTGGTCTGGAAAACTCTCTGCCGCTGGAAG<br>TCGTGCGTGGTGCAATGACCATCCGTGTTAATTCCCTGACGCGCGGTCATTCAGCTGTCCG<br>TCTGGTTGTCCTGGAAGCGCTGACCAACTTTCTGAATCACGGTATTACGCCGATCGTGCCG<br>CTGCGTGGTACCATTAGTGCATCCGGTGATCTGAGCCCGCTGTCTTATATTGCAGCTGCGA<br>TCTCTGGCCACCCGGACAGTAAAGTTCATGTGGTTCACGAGGGTAAAGAAAAAATCCTGT<br>ACGCCCGTGAAGCTATGGCGCTGTTCAACCTGGAACCGGTCGTGCTGGGCCCGAAAGAAG<br>GCCTGGGTCTGGTGAATGGTACGGCTGTTTCAGCGTCGATGGCCACCCTGGCACTGCATG<br>ATGCCCACATGCTGAGCCTGCTGAGCCAGTCTCTGACCGCGATGACGGTCGAAGCGATGG<br>TGGGCCATGCAGGTAGCTTTCATCCGTTCCTGCACGATGTGACCCGTCCGCACCCGACGC<br>AGATTGAAGTTGCAGGCAACATCCGCAAACTGCTGGAAGGTAGCCGTTTTGCGGTGCATC<br>ACGAAGAAGAAGTGAAAGTGAAAGATGACGAAGGCATTCTGCGCCAGGATCGTTATCCG<br>CTGCGTACCAGTCCGCAATGGCTGGGTCCGCTGGTCTCCGACCTGATTCATGCCCACGCA<br>GTGCTGACCATCGAAGCGGGTCAGAGTACCACGGATAACCCGCTGATTGACGTGGAAAA<br>TAAAACCTCTCATCACGGCGGTAACTTTCAAGCCGCAGCTGTTGCCAATACGATGGAAAA<br>AACGCGCCTGGGCCTGGCACAGATCGGTAAACTGAATTTCACCCAACTGACGGAAATGCT<br>GAACGCAGGCATGAATCGTGGTCTGCCGAGCTGCCTGGCAGCAGAAGATCCGAGTCTGTC<br>CTATCATTGTAAAGGCCTGGACATTGCAGCTGCGGCCTACACCTCTGAACTGGGTCATCT<br>GGCGAACCCGGTTACCACGCACGTCCAGCCGGCTGAAATGGCGAACCAAGCCGTGAATT<br>CCCTGGCACTGATCTCAGCTCGTCGCACCACGGAATCGAATGATGTCCTGAGCCTGCTGC<br>TGGCGACCCATCTGTATTGTGTTCTGCAGGCTATTGACCTGCGCGCGATCGAATTTGAATT<br>CAAAAAACAGTTTGGCCCGGCTATTGTGAGCCTGATCGATCAACACTTCGGCTCTGCCAT<br>GACCGGTAGTAACCTGCGTGACGAACTGGTGGAAAAAGTTAATAAAACGCTGGCCAAAC<br>GCCTGGAACAGACCAACAGTTACGATCTGGTGCCGCGTTGGCATGACGCATTTTCCTTCG<br>CAGCTGGTACGGTTGTCGAAGTTCTGAGTTCCACCTCACTGTCGCTGGCGGCCGTCAATGC<br>CTGGAAAGTGGCAGCTGCGGAAAGTGCAATTTCCCTGACCCGCCAAGTGCGTGAAACGTT<br>TTGGTCAGCAGCATCGACGTCATCGCCGGCACTGAGCTATCTGTCTCCGCGCACCCAAAT<br>TCTGTACGCTTTTGTTCGTGAAGAACTGGGCGTCAAAGCGCGTCGCGGCGATGTTTTCCTG<br>GGTAAACAGGAAGTGACCATCGGTAGTAATGTTTCCAAAATCTATGAAGCTATCAAAAGC<br>GGTCGTATCAATAATGTGCTGCTGAAAATGCTGGCATAA
***Fj*TAL**	ATGAACACCATCAACGAATATCTGAGCCTGGAAGAATTTGAAGCCATTATCTTTGGCAAT<br>CAGAAAGTGACCATTAGTGATGTTGTTGTGAATCGCGTTAACGAGAGCTTTAACTTTCTG<br>AAAGAATTTAGCGGCAACAAAGTGATCTATGGTGTGAATACCGGTTTTGGTCCGATGGCA<br>CAGTATCGTATTAAAGAAAGCGATCAGATTCAGCTGCAGTATAATCTGATTCGTAGCCAT<br>AGCAGCGGCACCGGTAAACCGCTGAGTCCGGTTTGTGCAAAAGCAGCAATTCTGGCACGT<br>CTGAATACCCTGAGTCTGGGTAATAGCGGTGTTCATCCGAGCGTTATTAATCTGATGAGC<br>GAACTGATCAACAAAGATATCACACCGCTGATTTTTGAACATGGTGGTGTTGGTGCAAGC<br>GGTGATCTGGTTCAGCTGAGCCATCTGGCACTGGTTCTGATTGGTGAAGGTGAAGTTTTCT<br>ATAAAGGTGAACGTCGTCCGACACCGGAAGTTTTTGAAATTGAAGGTCTGAAACCGATCC<br>AGGTGGAAATTCGCGAAGGTCTGGCCCTGATTAATGGCACCAGCGTTATGACCGGTATTG<br>GTGTTGTTAATGTGTACCATGCAAAAAAACTGCTGGATTGGAGCCTGAAAAGCAGCTGTG<br>CAATTAATGAACTGGTTCAGGCATATGATGATCACTTTAGCGCAGAACTGAATCAGACCA<br>AACGTCATAAAGGTCAGCAAGAAATTGCACTGAAAATGCGTCAGAATCTGAGCGATAGC<br>ACCCTGATTCGCAAACGTGAAGATCATCTGTATAGCGGTGAAAACACCGAAGAAATCTTC<br>AAAGAAAAAGTGCAAGAGTATTATAGCCTGCGTTGTGTTCCGCAGATTCTGGGTCCGGTT<br>CTGGAAACCATTAACAATGTTGCAAGCATTCTGGAAGATGAATTTAACAGCGCAAACGAT<br>AACCCGATCATCGATGTTAAAAACCAGCATGTTTATCACGGTGGCAATTTTCATGGTGATT<br>ATATCAGCCTGGAAATGGATAAACTGAAAATCGTGATTACCAAACTGACCATGCTGGCAG<br>AACGTCAGCTGAATTATCTGCTGAATAGCAAAATTAACGAACTGCTGCCTCCGTTTGTTA<br>ATCTGGGCACCCTGGGTTTTAACTTTGGTATGCAGGGTGTTCAGTTTACCGCAACCAGCAC<br>CACCGCAGAAAGCCAGATGCTGAGCAATCCGATGTATGTTCATAGCATTCCGAACAATAA<br>TGATAACCAGGATATTGTTAGCATGGGCACCAATAGCGCAGTTATTACCAGCAAAGTTAT<br>CGAAAATGCCTTTGAAGTTCTGGCCATTGAAATGATTACCATTGTTCAGGCGATTGATTAT<br>CTGGGCCAGAAAGATAAAATCAGCAGCGTTAGCAAAAAATGGTATGATGAAATCCGCAA<br>CATCATCCCGACCTTTAAAGAAGATCAGGTGATGTATCCGTTCGTGCAGAAAGTAAAAGA<br>CCACCTGATTAACAATTAA

***At*4CL**

ATGGCGCCACAAGAACAAGCAGTTTCTCAGGTGATGGAGAAACAGAGCAACAACAACAA
CAGTGACGTCATTTTCCGATCAAAGTTACCGGATATTTACATCCCGAACCACCTATCTCTC
CACGACTACATCTTCCAAAACATCTCCGAATTCGCCACTAAGCCTTGCCTAATCAACGGA
CCAACCGGCCACGTGTACACTTACTCCGACGTCCACGTCATCTCCCGCCAAATCGCCGCC
AATTTTCACAAACTCGGCGTTAACCAAAACGACGTCGTCATGCTCCTCCTCCCAAACTGTC
CCGAATTCGTCCTCTCTTTCCTCGCCGCCTCCTTCCGCGGCGCAACCGCCACCGCCGCAAA
CCCTTTCTTCACTCCGGCGGAGATAGCTAAACAAGCCAAAGCCTCCAACACCAAACTCAT
AATCACCGAAGCTCGTTACGTCGACAAAATCAAACCACTTCAAAACGACGACGGAGTAG
TCATCGTCTGCATCGACGACAACGAATCCGTGCCAATCCCTGAAGGCTGCCTCCGCTTCA
CCGAGTTGACTCAGTCGACAACCGAGGCATCAGAAGTCATCGACTCGGTGGAGATTTCAC
CGGACGACGTGGTGGCACTACCTTACTCCTCTGGCACGACGGGATTACCAAAAGGAGTGA
TGCTGACTCACAAGGGACTAGTCACGAGCGTTGCTCAGCAAGTCGACGGCGAGAACCCG
AATCTTTATTTCCACAGCGATGACGTCATACTCTGTGTTTTGCCCATGTTTCATATCTACGC
TTTGAACTCGATCATGTTGTGTGGTCTTAGAGTTGGTGCGGCGATTCTGATAATGCCGAAG
TTTGAGATCAATCTGCTATTGGAGCTGATCCAGAGGTGTAAAGTGACGGTGGCTCCGATG
GTTCCGCCGATTGTGTTGGCCATTGCGAAGTCTTCGGAGACGGAGAAGTATGATTTGAGC
TCGATAAGAGTGGTGAAATCTGGTGCTGCTCCTCTTGGTAAAGAACTTGAAGATGCCGTT
AATGCCAAGTTTCCTAATGCCAAACTCGGTCAGGGATACGGAATGACGGAAGCAGGTCC
AGTGCTAGCAATGTCGTTAGGTTTTGCAAAGGAACCTTTTCCGGTTAAGTCAGGAGCTTGT
GGTACTGTTGTAAGAAATGCTGAGATGAAAATAGTTGATCCAGACACCGGAGATTCTCTT
TCGAGGAATCAACCCGGTGAGATTTGTATTCGTGGTCACCAGATCATGAAAGGTTACCTC
AACAATCCGGCAGCTACAGCAGAGACCATTGATAAAGACGGTTGGCTTCATACTGGAGAT
ATTGGATTGATCGATGACGATGACGAGCTTTTCATCGTTGATCGATTGAAAGAACTTATC
AAGTATAAAGGTTTTCAGGTAGCTCCGGCTGAGCTAGAGGCTTTGCTCATCGGTCATCCT
GACATTACTGATGTTGCTGTTGTCGCAATGAAAGAAGAAGCAGCTGGTGAAGTTCCTGTT
GCATTTGTGGTGAAATCGAAGGATTCGGAGTTATCAGAAGATGATGTGAAGCAATTCGTG
TCGAAACAGGTTGTGTTTTACAAGAGAATCAACAAAGTGTTCTTCACTGAATCCATTCCTA
AAGCTCCATCAGGGAAGATATTGAGGAAAGATCTGAGGGCAAAACTAGCAAATGGATTG
TGA

***Gm*4CL**

ATGGCGCCTTCGCCGCAAGAAATCATTTTTCGCTCGCCACTTCCGGATATTCCGATCCCGA
CACATTTACCGCTCTATTCCTACTGCTTTCAGAATCTTTCACAGTTCCATGATCGCCCTTGT
CTTATTGACGGAGACACCGGCGAAACGCTGACATATGCAGACGTGGACCTGGCGGCCCG
CCGTATTGCGAGCGGCCTGCATAAGATTGGTATCAGACAAGGTGATGTTATTATGCTAGT
CCTCCGGAACTGTCCTCAATTCGCCTTAGCCTTTCTGGGAGCGACACACCGTGGGGCGGT
AGTGACCACTGCAAATCCCTTTTATACGCCCGCGGAGTTAGCCAAACAGGCGACAGCAAC
CAAAACCCGCCTGGTGATTACCCAAAGCGCCTACGTGGAAAAGATTAAAAGCTTTGCTGA
CAGCAGTAGCGACGTAATGGTTATGTGCATCGACGATGACTTTTCCTATGAAAACGATGG
TGTTCTTCACTTCAGCACACTAAGTAACGCCGATGAGACGGAAGCCCCAGCGGTGAAGAT
TAACCCTGATGAGCTGGTAGCCCTGCCATTTAGTTCAGGGACGTCTGGCCTACCAAAAGG
CGTGATGCTTAGCCACAAAAACCTGGTGACCACAATTGCCCAACTGGTTGATGGCGAGAA
CCCGCACCAGTATACACATTCTGAAGATGTGCTATTGTGCGTGCTGCCTATGTTTCATATC
TACGCCCTGAACTCCATCCTGTTGTGCGGTATCCGTTCAGGCGCCGCGGTGCTCATTCTGC
AGAAATTCGAAATTACCACTCTGCTGGAACTAATCGAGAAATACAAGGTGACGGTGGCC
AGTTTCGTTCCTCCGATTGTCTTAGCCTTGGTTAAATCAGGCGAGACACACCGATATGATC
TGAGTTCCATTCGCGCAGTAGTTACGGGCGCAGCCCCGTTAGGCGGAGAACTGCAAGAGG
CCGTCAAAGCACGCCTGCCTCACGCAACCTTTGGCCAGGGCTATGGTATGACTGAAGCAG
GCCCGCTGGCGATTAGCATGGCCTTTGCCAAAGTACCGTCGAAAATCAAGCCAGGGGCGT
GCGGTACCGTAGTGCGCAATGCGGAGATGAAAATTGTAGACACCGAAACCGGCGATAGT
CTACCGCGAAATAAACACGGCGAAATTTGCATTATTGGCACTAAAGTGATGAAAGGGTAT
CTCAACGACCCGGAAGCCACTGAACGCACCGTCGATAAAGAAGGCTGGTTACACACCGG
AGATATTGGTTTTATTGATGATGACGATGAATTATTCATTGTTGATCGCTTAAAAGAACTA
ATCAAATACAAAGGTTTTCAAGTGGCCCCGGCCGAACTGGAAGCCCTGCTGATTGCACAC
CCAAATATTTCGGATGCAGCCGTTGTGGGGATGAAAGATGAAGCTGCTGGTGAAATACCC
GTGGCATTTGTCGTGCGAAGTAATGGCAGCGAAATTGCCGAGGATGAGATCAAAAAGTA
TATTAGCCAGCAAGTGGTGTTTTACAAACGTATCTGTCGCGTGTTTTTCACCGACAGTATT
CCTAAGGCGCCGTCTGGCAAGATTCTGCGTAAAGTTCTCACGGCGCGCCTGAACGAAGGT
CTGGTTGTGGCCAATTA

***Vv*4CL**

ATGATTAGTATTGAAACGCAAAACCCGGATGTTAGCAACCTGGACACCTCGCACTCTATT
CCGAAAATGGCAAACCGTATTGATGACCATGTGTTTCGTTCTAAACTGCCGGAAATTCCG
ATCAGTAACCATCTGCCGCTGCACACGTATTGCTTCGAAAATTACTCGCAGTTTGCAGACC
GTCCGTGTCTGATTGTTGGCTCGACGAACAAAACCTATAGCTTCGCTGAAACCCATCTGAT
CTCTCGCAAAGTGGGCGCAGGTTTTGCTCACCTGGGTCTGAAACAGGGCGATGTGGTTAT
GATTCTGCTGCAAAATTGCGCGGAATTTGCCTTCAGCTTTCTGGGTGCGTCTATGGTTGGC
GCCGTCACCACGACCGCAAACCCGTTCTACACGTCCGCGGAAATCTTCAAACAGCTGAAC
GCATCAAAAGCTAAAATCGTCGTGACCCAGGCGCAATATGTGGATAAACTGCGCGACTAC

CCGGATGGTCAAGTTGCCAAAATTGGCGAAGGTTTCACGGTCATTACCATCGATGACCCG
CCGGAAAACTGTATGCATTTTAGTGTTGTCTCCGAAGCGAACGAAAGCGAACTGCCGGAA
GTCTCAATTAATTCGGATGACCCGGTGGCCCTGCCGTTTAGCTCTGGTACGACCGGCCTGC
CGAAAGGCGTGGTTCTGACGCACAAATCACTGATCACCTCGGTCGCCCAGCAAGTGGATG
GTGAAAACCCGAATCTGCATCTGACCCCGGATGACGTCGTGCTGTGCGTGCTGCCGCTGT
TCCACATTTATAGCCTGAACTCTGTTCTGCTGTGTAGTCTGCGTGCAGGTGCAGCAGTGCT
GCTGATGCAGAAATTTGAAATTGGTACCCTGCTGGAACTGATCCAACGTTACCGCGTGAG
CGTTGCAGCTGTTGTCCCGCCGCTGGTTCTGGCACTGGCTAAAAATCCGATGGTGGAATC
GTTTGATCTGAGTTCCATCCGTGTGGTTCTGAGCGGTGCAGCACCGCTGGGCAAAGAACT
GGAAGCAGCTCTGCGTTCCCGCGTTCCGCAGGCAGTCCTGGGCCAAGGTTATGGCATGAC
GGAAGCAGGCCCGGTGCTGTCAATGTGCCTGGGTTTCGCTAAACAGCCGTTTCCGACGAA
ATCAGGTTCGTGTGGCACCGTCGTGCGTAACGCGGAACTGAAAGTTGTGGATCCGGAAAC
CGGTTGCTCCCTGGGCCGTAATCAGCCGGGTGAAATTTGTATCCGCGGCCAGCAAATTAT
GAAAGGTTATCTGAATGATCCGGAAGCGACGGCCTCTACCATTGACGTTGATGGCTGGCT
GCATACCGGTGACATCGGCTACGTGGATGACGATGAAGAAGTGTTCATTGTTGATCGCGT
CAAAGAACTGATCAAATTCAAAGGTTTTCAGGTTCCGCCGGCAGAACTGGAAGCTCTGCT
GGTGTCTCACCCGTCCATTGCCGATGCGGCCGTGGTTCCGCAAAAAGACGATGTTGCTGG
CGAAGTCCCGGTGGCGTTCGTCGTGCGTTCTAACGGTTTTGAACTGACCGAAGAAGCAGT
GAAAGAATTCATCAGTAAACAGGTTGTCTTTTATAAACGCCTGCATAAAGTGTACTTTGTT
CACGCGATTCCGAAAAGCCCGTCTGGCAAAATCCTGCGTAAAGATCTGCGCGCGAAACTG
GCCGAAAAAACCCCGGAACCGAACGGCGGTGGCGGTAGTGGCGGTGGCGGTTCCGGCGG
TGGCGGTTCACTGGTGGGTGCCCTGATGCATGTTATGCAGAAACGTAGCCGTGCTATCCA
CTCCTCAGATGAAGGTGAAGACCAGGCAGGTGACGAAGATGAAGACTAA

***Pc*4CL**

ATGGGAGACTGTGTAGCACCCAAAGAAGACCTTATTTTCCGATCGAAACTCCCTGATATT
TACATCCCGAAACACCTTCCGTTACATACTTATTGTTTCGAAAACATCTCGAAAGTTGGCG
ACAAGTCCTGTTTAATAAATGGCGCTACAGGCGAAACGTTCACTTATTCCCAAGTTGAGC
TCCTTTCCAGGAAAGTTGCATCAGGGTTAAACAAACTCGGCATTCAACAGGGCGATACCA
TCATGCTTTTGCTCCCTAACTCCCCTGAGTATTTTTTCGCTTTCTTAGGCGCATCGTATCGT
GGTGCAATTTCTACTATGGCCAATCCGTTTTTCACTTCTGCTGAGGTGATCAAACAGCTCA
AAGCATCCCAAGCTAAGCTCATAATTACGCAAGCTTGTTACGTAGACAAAGTGAAAGACT
ACGCAGCAGAGAAAAATATACAGATCATTTGCATCGATGATGCTCCTCAGGATTGTTTAC
ATTTCTCCAAACTTATGGAAGCTGATGAATCAGAAATGCCTGAGGTTGTGATCAATTCAG
ACGATGTCGTCGCGTTACCTTACTCATCGGGTACTACAGGACTACCGAAAGGTGTTATGTT
GACACACAAAGGACTTGTTACTAGCGTGGCACAACAAGTTGATGGAGACAATCCGAATTT
ATATATGCATAGCGAGGATGTGATGATCTGCATATTGCCTTTGTTTCATATTTATTCGCTT
AACGCGGTGTTGTGCTGTGGACTCAGAGCAGGGGTGACGATCTTGATTATGCAGAAATTT
GATATTGTGCCATTTTTGGAACTGATACAGAAATATAAAGTTACAATTGGACCGTTTGTGC
CACCAATTGTGTTGGCAATTGCGAAAAGTCCAGTGGTGGATAAATATGACTTGTCGTCGG
TGAGGACGGTTATGTCTGGAGCTGCTCCGTTAGGGAAGGAGCTTGAAGATGCTGTTAGAG
CTAAGTTTCCTAATGCCAAACTTGGTCAGGGATATGGAATGACAGAGGCAGGGCCAGTTT
TAGCAATGTGCCTGGCGTTTGCAAAGGAACCATACGAGATCAAATCGGGTGCCTGTGGAA
CTGTTGTGAGGAATGCTGAAATGAAAATTGTGGATCCTGAGACCAACGCCTCTCTTCCAC
GAAACCAACGCGGAGAGATTTGCATTCGAGGTGACCAAATTATGAAAGGCTACCTCAAT
GATCCTGAATCAACAAGGACAACAATAGACGAAGAAGGCTGGTTGCACACAGGAGATAT
AGGCTTCATTGACGACGATGATGAGCTATTTATTGTTGATAGACTTAAGGAAATAATCAA
ATACAAAGGCTTCCAGGTTGCCCCTGCTGAACTTGAAGCTCTGCTACTTACTCATCCTACC
ATTTCCGATGCTGCAGTTGTTCCCATGATAGATGAGAAAGCAGGAGAGGTGCCTGTGGCT
TTTGTTGTGAGAACAAACGGTTTCACCACCACTGAGGAAGAAATCAAGCAATTCGTCTCG
AAACAGGTGGTGTTCTACAAGAGAATATTTCGTGTATTTTTTGTTGATGCAATTCCGAAAT
CACCATCTGGAAAGATTCTTCGAAAGGACTTGAGAGCAAAAAATAGCATCCGGTGATCTTC
CCAAATAA

***At*CHS**

ATGGTGATGGCTGGTGCTTCTTCTTTGGATGAGATCAGACAGGCTCAGAGAGCTGATGGA
CCTGCAGGCATCTTGGCTATTGGCACTGCTAACCCTGAGAACCATGTGCTTCAGGCGGAG
TATCCTGACTACTACTTCCGCATCACCAACAGTGAACACATGACCGACCTCAAGGAGAAG
TTCAAGCGCATGTGCGACAAGTCGACAATTCGGAAACGTCACATGCATCTGACGGAGGA
ATTCCTCAAGGAAAACCCACACATGTGTGCTTACATGGCTCCTTCTCTGGACACCAGACA
GGACATCGTGGTGGTCGAAGTCCCTAAGCTAGGCAAAGAAGCGGCAGTGAAGGCCATCA
AGGAGTGGGGCCAGCCCAAGTCAAAGATCACTCATGTCGTCTTCTGCACTACCTCCGGCG
TCGACATGCCTGGTGCTGACTACCAGCTCACCAAGCTTCTTGGTCTCCGTCCTTCCGTCAA
GCGTCTCATGATGTACCAGCAAGGTTGCTTCGCCGGCGGTACTGTCCTCCGTATCGCTAAG
GATCTCGCCGAGAACAACCGTGGAGCACGTGTCCTCGTTGTCTGCTCTGAGATCACAGCC
GTTACCTTCCGTGGTCCCTCTGACACCCACCTTGACTCCCTCGTCGGTCAGGCTCTTTTCA
GTGATGGCGCCGCCGCACTCATTGTGGGGTCGGACCCTGACACATCTGTCGGAGAGAAAC
CCATCTTTGAGATGGTGTCTGCCGCTCAGACCATCCTTCCAGACTCTGATGGTGCCATAGA
CGGACATTTGAGGGAAGTTGGTCTCACCTTCCATCTCCTCAAGGATGTTCCCGGCCTCATC

TCCAAGAACATTGTGAAGAGTCTAGACGAAGCGTTTAAACCTTTGGGGATAAGTGACTGG
AACTCCCTCTTCTGGATAGCCCACCCTGGAGGTCCAGCGATCCTAGACCAGGTGGAGATA
AAGCTAGGACTAAAGGAAGAGAAGATGAGGGCGACACGTCACGTGTTGAGCGAGTATGG
AAACATGTCGAGCGCGTGCGTTCTCTTCATACTAGACGAGATGAGGAGGAAGTCAGCTAA
GGATGGTGTGGCCACGACAGGAGAAGGGTTGGAGTGGGGTGTCTTGTTTGGTTTCGGACC
AGGTCTCACTGTTGAGACAGTCGTCTTGCACAGCGTTCCTCTCTAA

_Cm_CHS

ATGGCTACGGTCCAAGAAATCCGCAACGCTCAACGCGCAGATGGTCCGGCGACGGTCCTG
GCAATCGGCACGGCAACCCCGGCTCATAGCGTGAACCAGGCAGATTATCCGGACTATTAC
TTTCGTATTACCAAATCTGAACACATGACGGAACTGAAAGAAAAATTCAAACGTATGTGC
GATAAAAGTATGATTAAAAAACGCTACATGTACCTGACCGAAGAAATCCTGAAAGAAAA
CCCGAATATGTGTGCCTACATGGCACCGAGCCTGGATGCGCGCCAGGACATTGTGGTTGT
CGAAGTTCCGAAACTGGGTAAAGAAGCGGCCACCAAAGCCATCAAAGAATGGGGCCAAC
CGAAATCAAAAATTACGCACCTGATCTTTTGCACCACGTCGGGTGTGGATATGCCGGGTG
CAGACTATCAGCTGACCAAACTGCTGGGTCTGCGTCCGAGCGTTAAACGCTTTATGATGT
ACCAGCAAGGCTGCTTCGCAGGCGGTACGGTCCTGCGTCTGGCTAAAGATCTGGCGGAAA
ACAATAAAGGTGCTCGCGTTCTGGTGGTTTGTAGTGAAATTACCGCTGTCACGTTTCGTGG
TCCGGCGGATACCCATCTGGACTCCCTGGTTGGCCAGGCCCTGTTCGGCGATGGTGCAGC
TGCGGTTATCGTCGGCGCAGATCCGGACACGAGTGTGGAACGTCCGCTGTATCAGCTGGT
TTCAACCTCGCAAACGATTCTGCCGGATTCCGACGGTGCGATCGATGGCCATCTGCGCGA
AGTGGGTCTGACCTTTCACCTGCTGAAAGACGTTCCGGGCCTGATTTCAAAAAACATCGA
AAAAAGCCTGTCTGAAGCCTTTGCACCGGTTGGTATTTCGGATTGGAGCTCTATTTTCTGG
ATCGCACATCCGGGCGGTCCGGCAATCCTGGACCAGGTGGAAAGCAAACTGGGTCTGAA
AGAAGAAAAACTGAAAGCTACCCGTCAAGTCCTGTCTGAATACGGCAATATGAGTTCCGC
GTGTGTGCTGTTCATTCTGGATGAAATGCGCAAAAAAATCTGCCGAAGAAGCTAAAGCGAC
CACGGGCGAAGGTCTGGATTGGGGCGTGCTGTTTGGTTTCGGTCCGGGTCTGACCGTCGA
AACGGTCGTGCTGCACAGTGTGCCGATCAAAGCGGGCGGTGGCGGTTCCGGCGGTGGTG
GTAGTGGTGGTGGTGGCTCTCCGCCGCCGGCCCTGCCGCCGAAACGTCGTCGCTAA

_Ph_CHS

ATGGTGACAGTCGAGGAGTATCGTAAGGCACAACGTGCTGAAGGTCCAGCCACTGTCATG
GCCATTGGAACAGCCACACCTTCAAACTGTGTTGATCAAAGCACTTACCCTGATTTTTATT
TTCGTATCACTAACAGTGAGCACAAGACTGATCTTAAGGAGAAATTTAAGCGCATGTGTG
AAAAATCAATGATTAAGAAAAGGTACATGCACTTAACAGAGGAAATCTTGAAAGAGAAT
CCTAGTATGTGTGAATACATGGCACCTTCTCTTGATGCTAGGCAAGACATAGTGGTGGTT
GAAGTGCCCAAACTTGGCAAAGAGGCAGCTCAAAAGGCCATCAAGGAATGGGGCCAGCC
CAAGTCCAAAATTACCCATTTGGTCTTTTGCACAACCAGTGGTGTGGACATGCCTGGGTGT
GACTATCAACTCACTAAGCTACTTGGGCTTCGTCCATCGGTCAAGAGGCTTATGATGTACC
AACAAGGTTGCTTTGCTGGTGGCACGGTTCTTCGGTTAGCCAAGGACTTGGCTGAAAACA
ACAAGGGCGCTCGAGTCCTTGTTGTTTGTTCAGAAATCACCGCGGTCACTTTCCGTGGGCC
AAATGATACTCATTTGGATAGTTTAGTTGGCCAAGCACTTTTTGGTGATGGGGCAGGCGC
GATCATTATAGGTTCTGATCCAATTCCAGGGGTCGAAAGGCCTTTGTTCGAGCTCGTTTCA
GCAGCCCAAACTCTTCTCCCAGATAGCCATGGTGCTATTGATGGCCATCTCCGTGAAGTTG
GGCTTACATTCCACTTACTCAAAGATGTTCCTGGGCTGATCTCAAAAAATATTGAGAAGA
GCCTTGAGGAAGCATTCAAACCTTTGGGCATTTCTGATTGGAACTCTCTATTCTGGATTGC
TCATCCAGGTGGGCCTGCAATTTTGGACCAAGTTGAAATAAAGTTGGGCCTAAAGCCCGA
GAAACTTAAGGCTACAAGGAATGTGTTAAGTAACTATGGTAACATGTCAAGTGCTTGTGT
ACTGTTTATTTTGGATGAAATGAGAAAGGCCTCAGCCAAAGAAGGTTTAGGAACTACTGG
TGAAGGGCTTGAGTGGGGTGTTCTTTTTGGATTTGGGCCTGGGCTAACAGTTGAGACTGTT
GTCCTCCACAGTGTTGCTACTTAA

_At_CHI

ATGTCTTCATCCAACGCCTGCGCCTCTCCGTCACCGTTCCCCGCCGTCACGAAGCTTCATG
TAGACTCCGTCACGTTTGTACCGTCCGTCAAGTCACCGGCCTCCTCCAATCCATTATTCCT
CGGCGGCGCCGGTGTCCGAGGCCTTGATATCCAAGGTAAATTCGTGATCTTCACCGTCAT
TGGAGTATACCTAGAGGGTAACGCCGTTCCTTCTCTATCTGTCAAGTGGAAGGGAAAAAC
TACGGAGGAGCTAACAGAATCTATCCCGTTCTTCCGTGAAATAGTCACCGGTGCGTTTGA
GAAGTTTATCAAGGTGACAATGAAACTGCCGTTAACGGGACAACAATATTCGGAGAAAG
TGACGGAGAATTGTGTGGCTATATGGAAACAATTAGGGCTTTATACGGACTGTGAAGCTA
AAGCTGTGGAGAAGTTCTTGGAGATCTTCAAGGAAGAAACATTCCCTCCCGGTTCATCGA
TCCTCTTCGCTCTCTCCCCTACCGGCTCTCTTACGGTTGCGTTTTCGAAAGATGATAGTATC
CCTGAAACCGGGATCGCTGTGATCGAGAACAAATTGTTGGCGGAGGCGGTTCTGGAATCT
ATCATCGGGAAGAACGGTGTGTCACCTGGCACTAGGTTAAGTGTTGCAGAAAGATTATCT
CAGCTAATGATGAAGAACAAGGACGAAAAGGAAGTTAGTGATCACTCTGTTGAGGAAAA
ACTAGCCAAAGAGAACTGA

_Cm_CHI

ATGAATCCGTCGCCGTCTGTTACCGAACTGCAAGTGGAAAATGTCACCTTTACGCCGAGT
CTGCAACCGCCGGGCTCTACCAAATCGCATTTTCTGGGCGGTGCAGGTGAACGTGGCCTG
GAAATCGAAGGCAAATTTGTTAAATTCACCGCTATTGGTGTCTATCTGGAAGAAAACGCC

	GTGCCGCTGCTGGCAGGCAAATGGAAAGGCAAAACCGCCGGTGAACTGACGGAATCTGT<br>CGAATTTTTCCGCGATGTGGTTACCGGCCCGTTTGAAAAATTCATGAAAGTGACCATGAT<br>CCTGCCGCTGACGGGTGCGCAGTATTCAGAAAAAGTTGCTGAAAATTGCATGGCGATTTG<br>GAAATTTTTCGGCATCTACACCGATGCAGAAGCTAAAGCGATTGAAAAATTTACGGAAGT<br>GTTCAAAGACGAAATTTTTCCGCCGGGCAGCTCTATCCTGTTCACCCAAAGTTCCGGTTCG<br>CTGACGATTTCATTTTCGAAAGATGGCAGCATCCCGAAAGACGGTGTCGCGGTGATTGAA<br>AACAATCTGCTGAGCGAAGCCGTTCTGGAATCTATGATCGGTAAAAACGGCGTCAGTCCG<br>GCGGCCAAAAAATCCCTGGCCGAACGTCTGTCAGCACTGCTGAATGTTGCTTCCGACAAA<br>ATGAAAGGCGGTGGCGGCTCAGGTGGCGGTGGCTCTGGTGGCGGTGGTTCAGGCGTCAA<br>AGAAAGTCTGGTGTGA
***Ms*CHI**	ATGGCTGCATCAATCACCGCAATCACTGTGGAGAACCTTGAATACCCAGCGGTGGTTACC<br>TCTCCGGTCACCGGCAAATCATATTTCCTCGGTGGCGCTGGGGAGAGAGGATTGACCATT<br>GAAGGAAACTTCATCAAGTTCACTGCCATAGGTGTTTATTTGGAAGATATAGCAGTGGCT<br>TCACTAGCTGCCAAATGGAAGGGTAAATCATCTGAAGAGTTACTTGAGACCCTTGACTTT<br>TACAGAGACATCATCTCAGGTCCCTTTGAAAAGTTAATTAGAGGGTCAAAGATTAGGGAA<br>TTGAGTGGTCCTGAGTACTCAAGGAAGGTTATGGAGAACTGTGTGGCACACTTGAAATCA<br>GTTGGAACTTATGGAGATGCAGAAGCTGAAGCTATGCAAAAATTTGCTGAAGCTTTCAAG<br>CCTGTTAATTTTCCACCTGGTGCCTCTGTTTTCTACAGGCAATCACCTAATGGAATATTAG<br>GGCTTAGTTTCTCTCCGGATACAAGTATACCAGAAAAGGAGGCTGCACTCATAGAGAACA<br>AGGCAGTTTCATCAGCAGTGTTGGAGACTATGATCGGCGAGCACGCTGTTTCCCCTGATC<br>TTAAGCGCTGTTTAGCTGCAAGATTACCTGCGTTGTTGAACGAGGGTGCTTTCAAGATTGG<br>AAACTGA

Table S2. List of primers used in this study for cloning of the desired genes, colony PCR and sequencing.

Primer	Sequence[1]	Restriction Enzyme
***Fj*TAL_BamHI_Fwd**	AAAA**GGATCC***CATG*AACACCATCAACGAATATCTG	*Bam*HI
***Fj*TAL_HindIII_Rev**	AACCC**AAGCTT***TTA*ATTGTTAATCAGGTGGTC	*Hind*III
***Gm*4CL_BamHI_Fwd**	AAAAA**GGATCCC***ATG*GCGCCTTCGCCG	*Bam*HI
***Gm*4CL_EcoRI_Rev**	AAAAA**GAATTC***TAA*TTGGCCACAACCAGACC	*Eco*RI
***Vv*4CL_HindIII_Fwd**	AAAAA**AAGCTT***ATG*ATTAGTATTGAAACGCAA	*Hind*III
***Vv*4CL_NotI_Rev**	AAAAA**AAGCTT***ATG*ATTAGTATTGAAACGCAA	*Not*I
***Pc*4CL_EcoRI_Fwd**	AAAAA**GAATTC**T*ATG*GGAGACTGTGTAGCA	*Eco*RI
***Pc*4CL_NotI_Rev**	AAAAA**GCGGCCGC***TTA*TTTGGGAAGATCACCGG	*Not*I
***At*CHS_NdeI_Fwd**	AAAAA**CAT*ATG***GTGATGGCTGGTGCT	*Nde*I
***At*CHS_XhoI_ Rev**	AAAAA**CTCGAG***TTA*GAGAGGAACGCTGTGC	*Xho*I
***At*CHI_NdeI_Fwd**	AAAAA**CAT*ATG***TCTTCATCCAACGCCTG	*Nde*I
***At*CHI_XhoI_Rev**	AAAAA**CTCGAG***TCA*GTTCTCTTTGGCTAGTTT	*Xho*I
***Cm*CHI_NdeI_Fwd**	AAAAAA**CAT*ATG***AATCCGTCGCCG	*Nde*I
***Cm*CHI_XhoI_Rev**	AAAAA**CTCGAG***TCA*CACCAGACT	*Xho*I
***Ms*CHI _NdeI_Fwd**	AAAAAA**CAT*ATG***GCTGCATCAATCA	*Nde*I
***Ms*CHI_XhoI_Rev**	AAAAA**CTCGAG***TCA*GTTTCCAATCTTGAAAGC	*Xho*I
***At*4CL_NdeI_Fwd**	AAAAA**CATATG***ATG*GCGCCACAAGAACA	*Nde*I
***At*4CL_XhoI_Rev**	AAAAA**CTCGAG***TCA*CAATCCATTTGCTAGTTTTGC	*Xho*I
**MCS1_Fwd**	GGATCTCGACGCTCTCCCTT	-
**MCS1_Rev**	CGATTATGCGGCCGTGTAC	-
**MCS2_Fwd**	GTACACGGCCGCATAATCG	-
**T7term_Rev**	CTAGTTATTGCTCAGCGGT	-

[1] Start and stop codons in *italic*; restriction sites in **bold**. To maintain the sequence in frame, one base was occasionally added between the restriction site and the gene start codon.

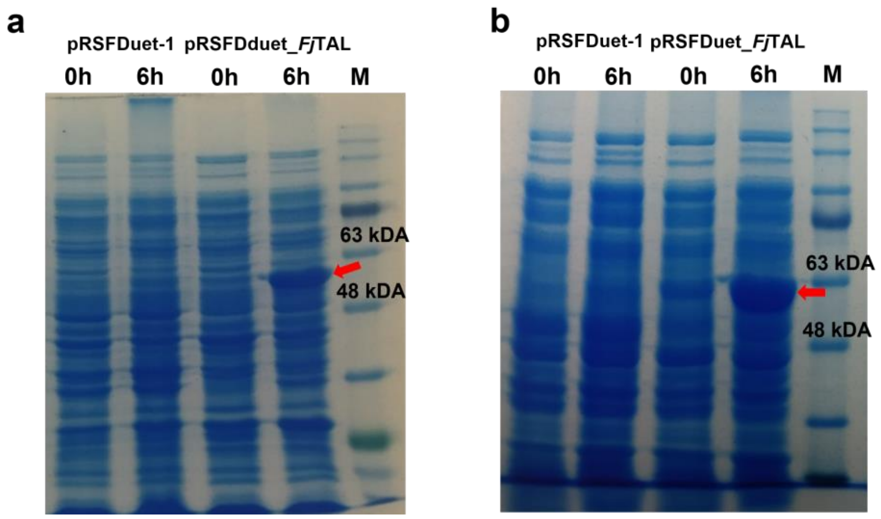

Fig. S1 Protein SDS-PAGE gels of soluble (a) and insoluble (b) protein fractions showing tyrosine ammonia-lyase from *Flavobacterium johnsoniae* (*Fj*TAL) expression in *E. coli* M-PAR-121 strain at time zero (0 h) of induction and after 6 h of induction. *Fj*TAL is expected around 58.26 kDa. Red arrows indicate the bands of interest. M: marker (NZYColour Protein Marker II – NZYTech).

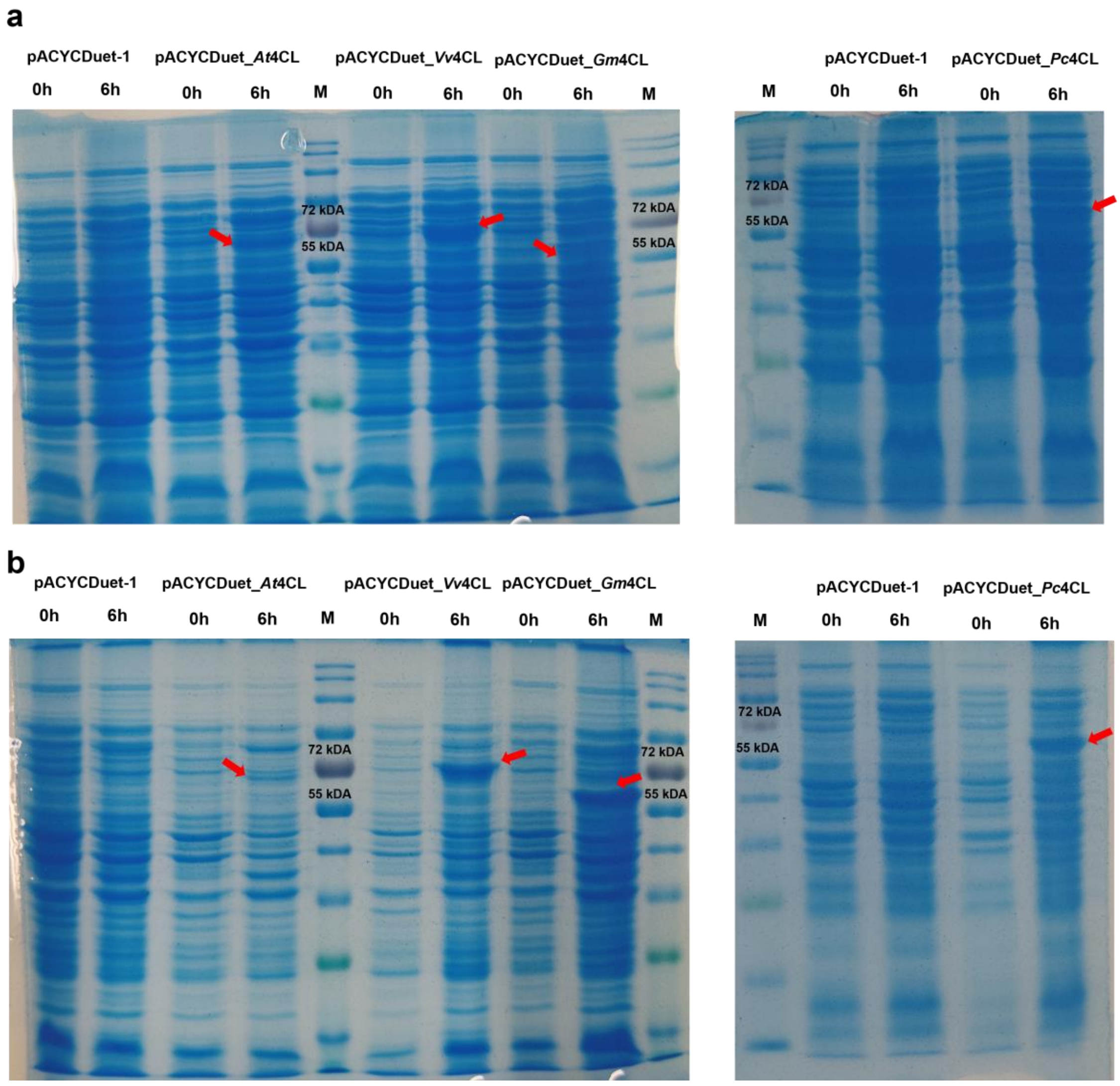

Fig. S2 Protein SDS-PAGE gels of soluble (a) and insoluble (b) protein fractions showing 4-coumarate-CoA ligase (4CL) from *Arabidopsis thaliana* (*At*4CL), 4CL from *Vitis vinifera* (*Vv*4CL), 4CL from *Glycine max* (*Gm*4CL), and 4CL from *Petroselinum crispum* (*Pc*4CL) expression in *E. coli* M-PAR-121 strain at time zero (0 h) of induction and after 6 h of induction. *At*4CL, *Vv*4CL, *Gm*4CL, *Pc*4CL are expected around 62.67 kDa, 69.25 kDa, 60.97 kDa, and 61.61 kDa respectively. Red arrows indicate the bands of interest. M: marker (Color Pre-stained Protein Standard, Broad Range (10-250 kDa) – NEB).

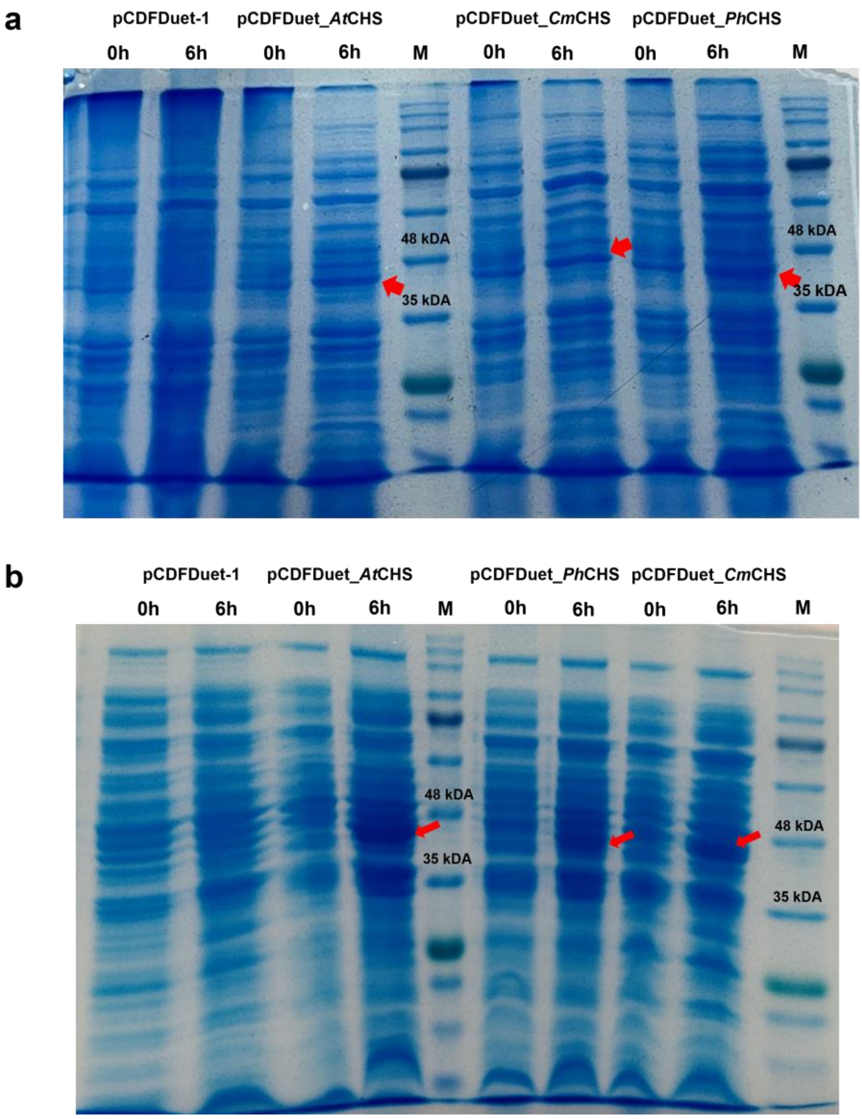

Fig. S3 Protein SDS-PAGE gels of soluble (a) and insoluble (b) protein fractions showing chalcone synthase (CHS) from *Arabidopsis thaliana* (*At*CHS), CHS from *Petunia hybrida* (*Ph*CHS), and CHS from *Cucurbita maxima* (*Cm*CHS) expression in *E. coli* M-PAR-121 strain at time zero (0 h) of induction and after 6 h of induction. *At*CHS, *Ph*CHS, and *Cm*CHS are expected around 43.12 kDa, 42.50 kDa and 44.81 kDa, respectively. Red arrows indicate the bands of interest. M: marker (NZYColour Protein Marker II – NZYTech).

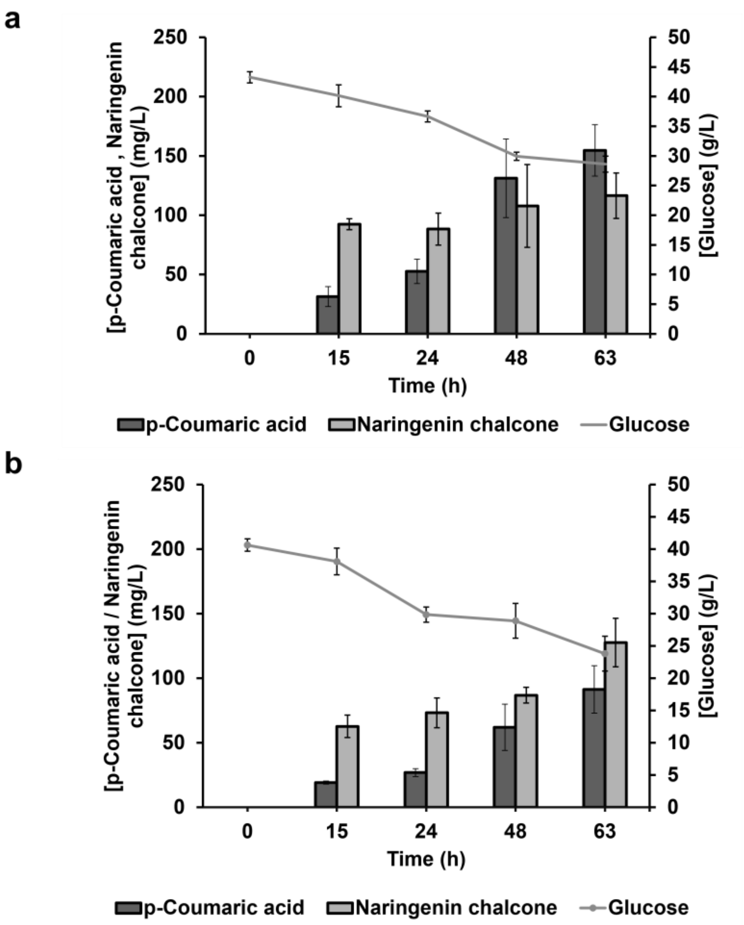

Fig. S4 Evaluation of the effect of metabolic burden reduction in the production of naringenin chalcone. a. Profile of naringenin chalcone and *p*-coumaric acid production and glucose consumption for the *E. coli* M-PAR-121 strain expressing pACYCDuet_*Fj*TAL and pRSFDuet_*At*4CL_*Cm*CHS. **b.** Profile of naringenin chalcone and *p*-coumaric acid production and glucose consumption for the *E. coli* M-PAR-121 strain expressing pACYCDuet_*Fj*TAL_*At*4CL and pRSFDuet_*Cm*CHS. Results correspond to the average of three independent experiments ± standard deviation.

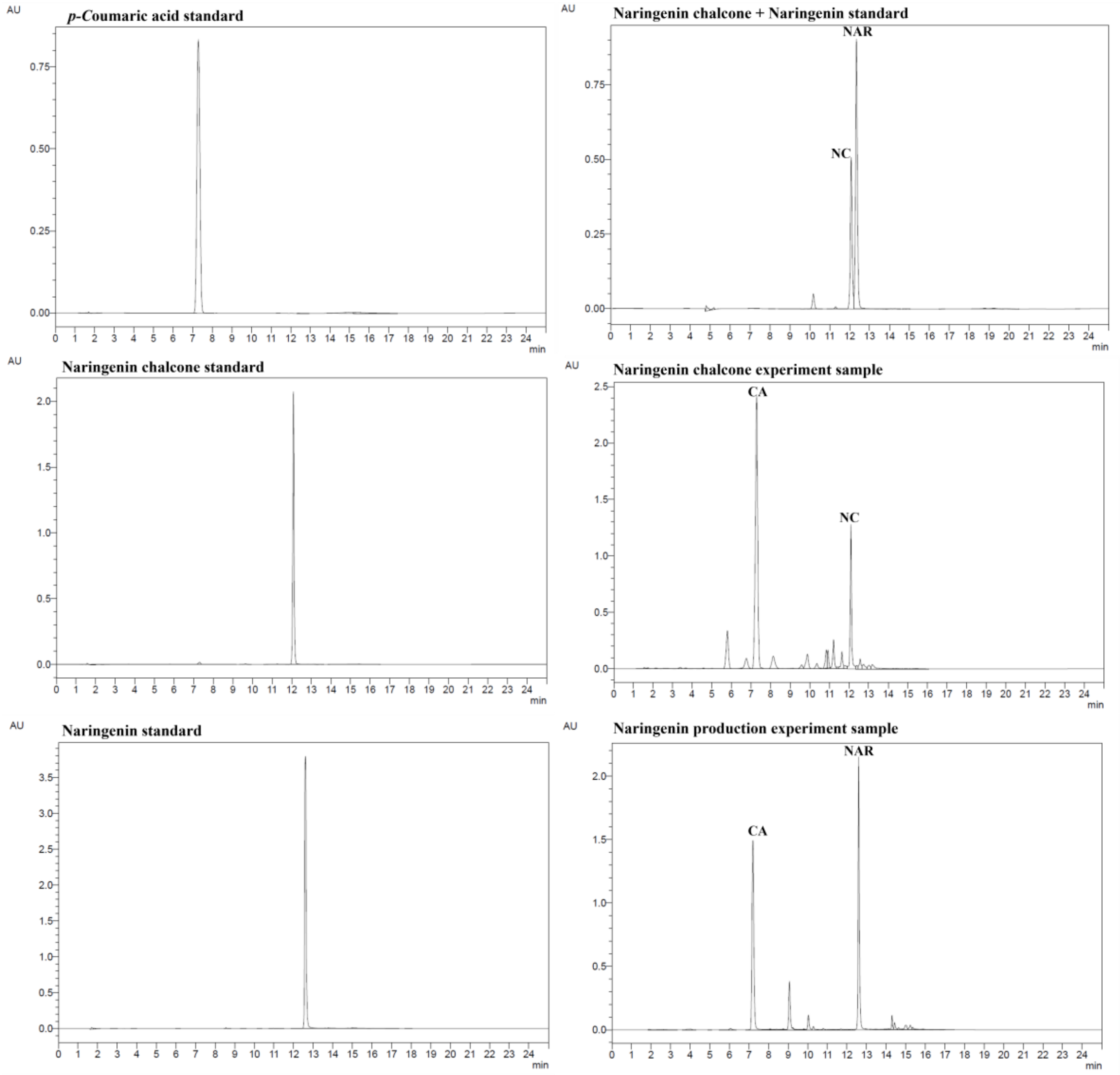

Fig. S5 Representative chromatograms of analytical standards (*p*-coumaric acid, naringenin chalcone, naringenin and the mixture of naringenin chalcone and naringenin standards) and samples from naringenin chalcone and naringenin production experiments with higher production levels. CA corresponds to *p*-coumaric acid. NC corresponds to naringenin chalcone. NAR corresponds to naringenin.

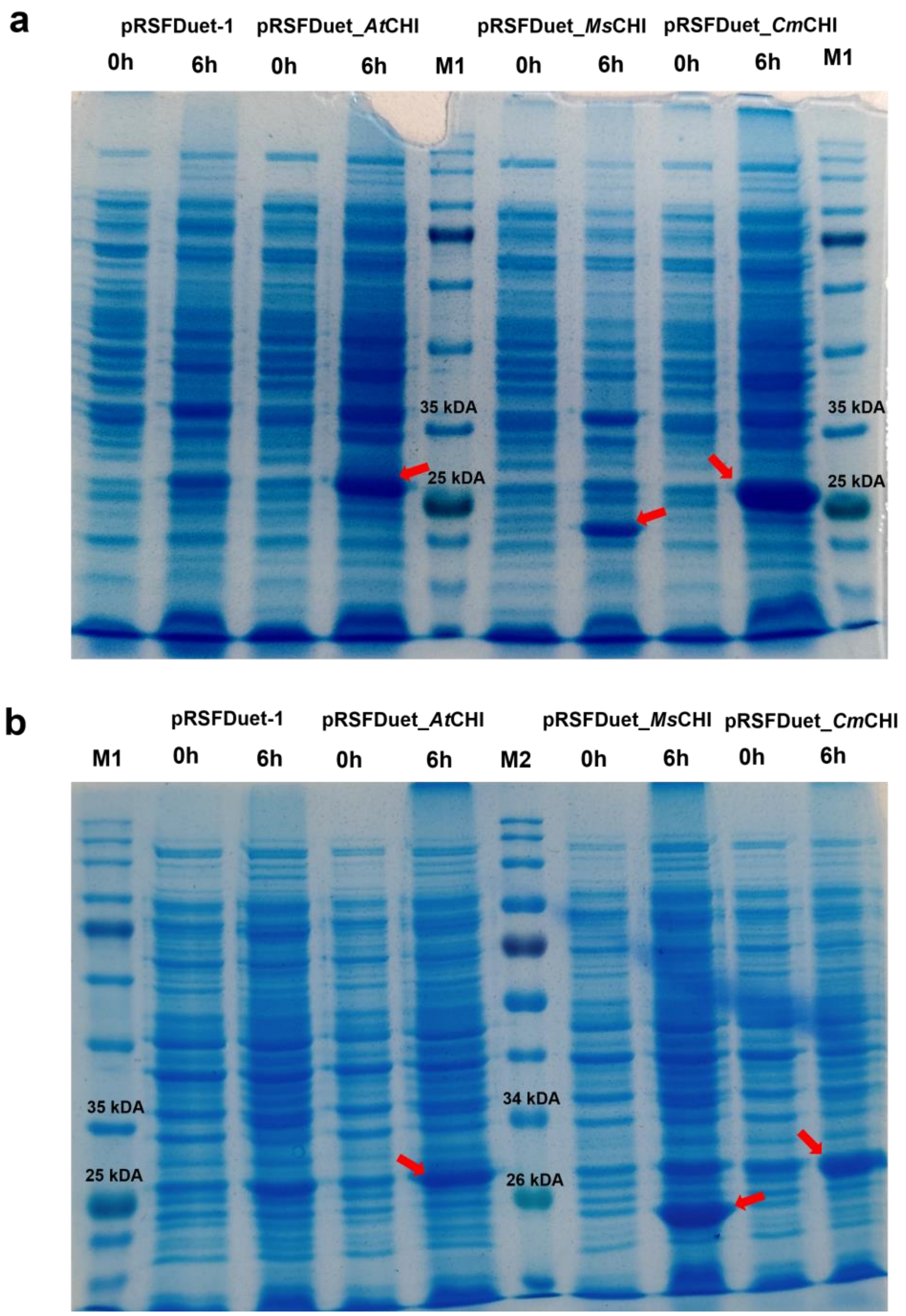

Fig. S6 Protein SDS-PAGE gels of soluble (a) and insoluble (b) protein fractions showing chalcone isomerase (CHI) from *Arabidopsis thaliana* (*At*CHI), CHI from *Medicago sativa* (*Ms*CHI), and CHI from *Cucurbita maxima* (*Cm*CHI) expression in *E. coli* M-PAR-121 strain at time zero (0 h) of induction and after 6 h of induction. *At*CHI, *Ms*CHI, and *Cm*CHI are expected around 26.60 kDa, 23.83 kDa and 25.60 kDa, respectively. Red arrows indicate the bands of interest. M1: marker 1 (NZYColour Protein Marker II – NZYTech); M2: marker 2 (Color Pre-stained Protein Standard, Broad Range (10-250 kDa) – NEB).

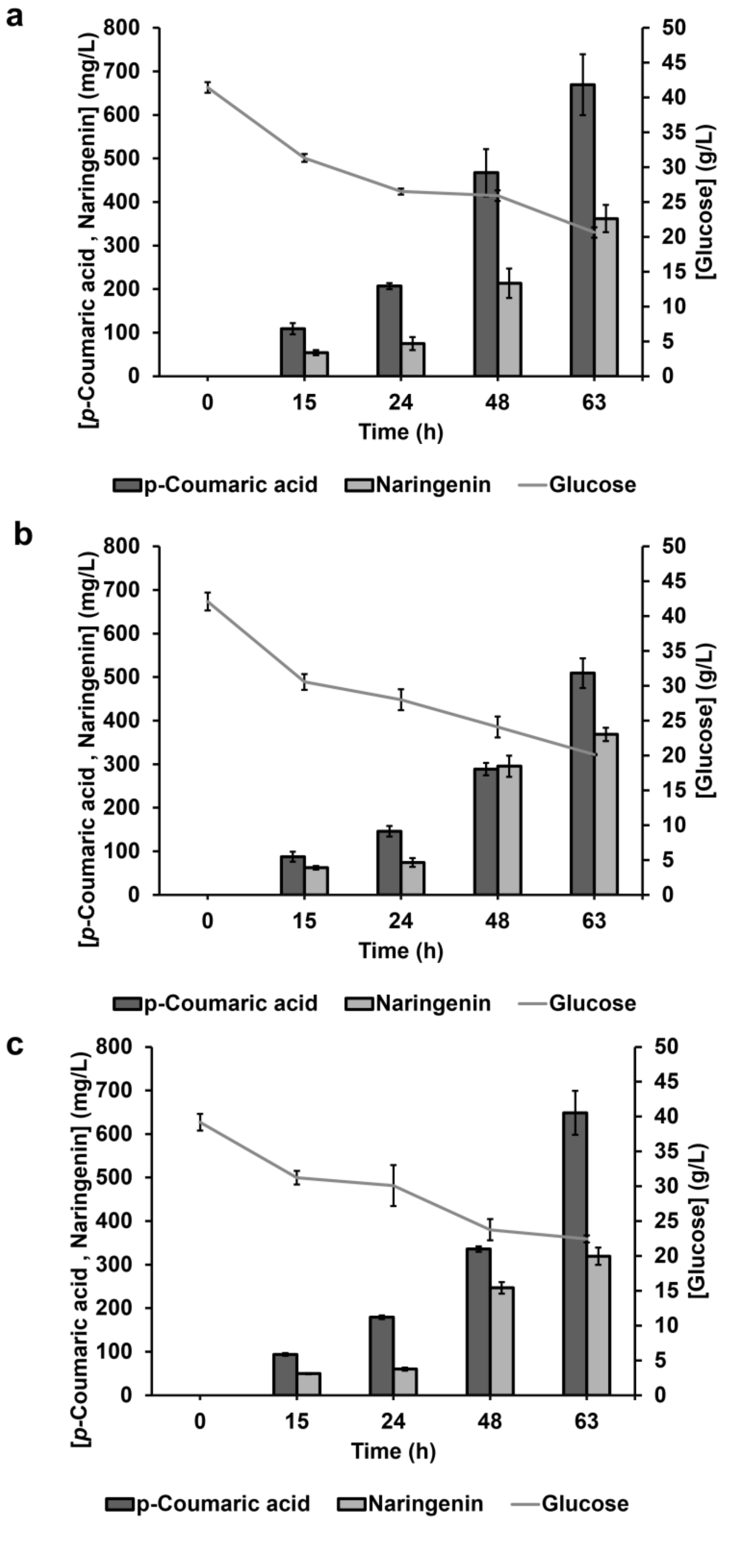

Fig. S7 Naringenin production by *Escherichia coli* M-PAR-121 expressing three different biosynthetic pathways after 63 h of fermentation. a. Profile of naringenin and *p*-coumaric acid production, and glucose consumption for the *E. coli* M-PAR-121 strain expressing pRSFDuet_*Fj*TAL_*Cm*CHS and pACYCDuet_*At*4CL_*At*CHI. **b.** Profile of naringenin and *p*-coumaric acid production and glucose consumption, for the *E. coli* M-PAR-121 strain expressing pRSFDuet_*Fj*TAL_*Cm*CHS and pACYCDuet_*At*4CL_*Cm*CHI. **c.** Profile of naringenin and *p*-coumaric acid production and glucose consumption, for the *E. coli* M-PAR-121 strain expressing pRSFDuet_*Fj*TAL_*Cm*CHS and pACYCDuet_*At*4CL_*Ms*CHI. Results correspond to the average of three independent experiments ± standard deviation.

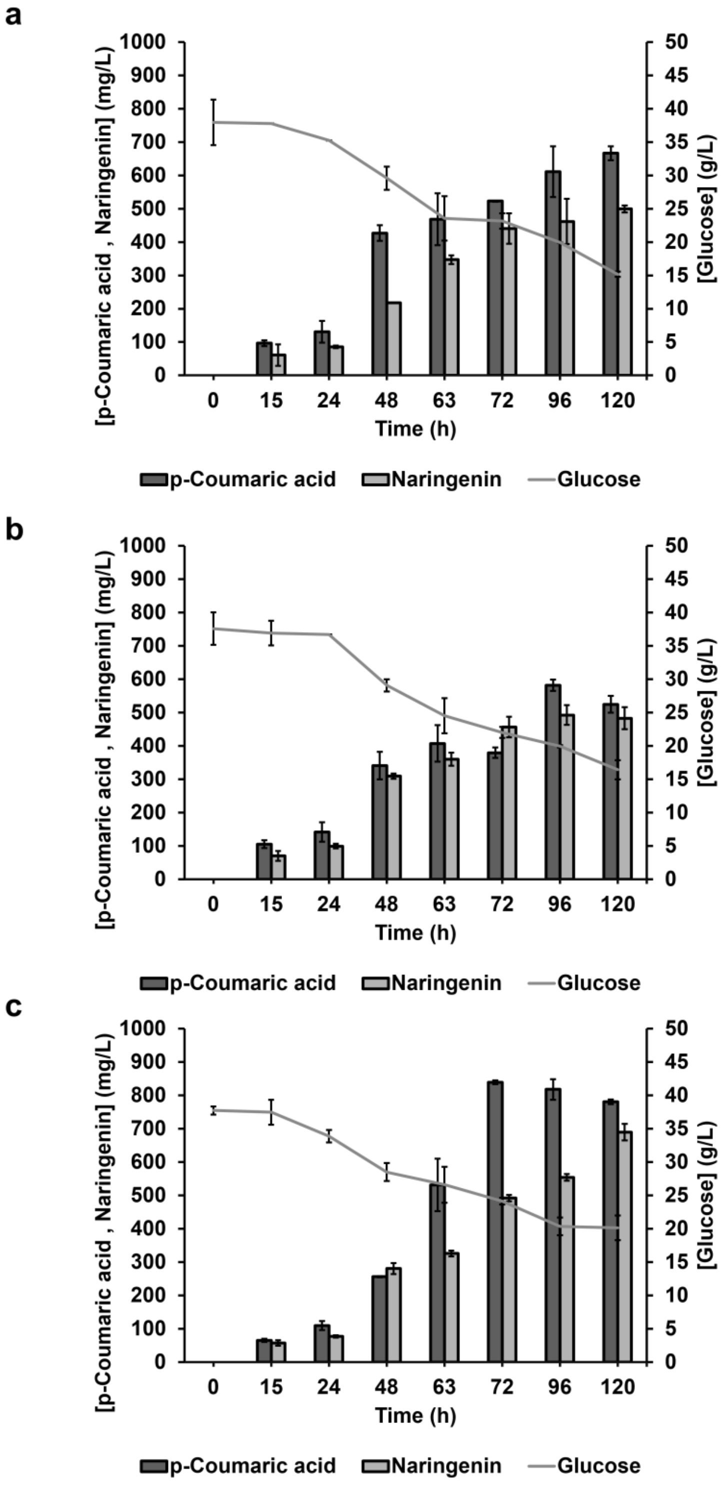

Fig. S8 Evaluation of fermentation time increase to 120 h in the production of naringenin. a. Profile of naringenin and *p*-coumaric acid production, and glucose consumption for the *E. coli* M-PAR-121 strain expressing pRSFDuet_*Fj*TAL_*Cm*CHS and pACYCDuet_*At*4CL_*At*CHI. **b.** Profile of naringenin and *p*-coumaric acid production and glucose consumption, for the *E. coli* M-PAR-121 strain expressing pRSFDuet_*Fj*TAL_*Cm*CHS and pACYCDuet_*At*4CL_*Cm*CHI. **c.** Profile of naringenin and *p*-coumaric acid production and glucose consumption, for the *E. coli* M-PAR-121 strain expressing pRSFDuet_*Fj*TAL_*Cm*CHS and pACYCDuet_*At*4CL_*Ms*CHI Results correspond to the average of three independent experiments ± standard deviation.
